# Exclusive and ultrasensitive detection of formaldehyde at room temperature using a flexible and monolithic chemiresistive sensor

**DOI:** 10.1038/s41467-021-25290-3

**Published:** 2021-08-16

**Authors:** Yong Kun Jo, Seong-Yong Jeong, Young Kook Moon, Young-Moo Jo, Ji-Wook Yoon, Jong-Heun Lee

**Affiliations:** 1 Department of Materials Science and Engineering, Korea University, Seoul, Republic of Korea; 2 Department of Information Materials Engineering, Jeonbuk National University, Jeonju, Republic of Korea

**Keywords:** Sensors and biosensors, Electronic properties and materials, Organic-inorganic nanostructures

## Abstract

Formaldehyde, a probable carcinogen, is a ubiquitous indoor pollutant, but its highly selective detection has been a long-standing challenge. Herein, a chemiresistive sensor that can detect ppb-level formaldehyde in an exclusive manner at room temperature is designed. The TiO_2_ sensor exhibits under UV illumination highly selective detection of formaldehyde and ethanol with negligible cross-responses to other indoor pollutants. The coating of a mixed matrix membrane (MMM) composed of zeolitic imidazole framework (ZIF-7) nanoparticles and polymers on TiO_2_ sensing films removed ethanol interference completely by molecular sieving, enabling an ultrahigh selectivity (response ratio > 50) and response (resistance ratio > 1,100) to 5 ppm formaldehyde at room temperature. Furthermore, a monolithic and flexible sensor is fabricated successfully using a TiO_2_ film sandwiched between a flexible polyethylene terephthalate substrate and MMM overlayer. Our work provides a strategy to achieve exclusive selectivity and high response to formaldehyde, demonstrating the promising potential of flexible gas sensors for indoor air monitoring.

## Abstract (web summary)

Formaldehyde, a probable carcinogen, is a ubiquitous indoor pollutant, but its ultraselective detection has been a long-standing challenge. Here, the authors develop a chemiresistive sensor that can detect ppb-level formaldehyde in an exclusive manner at room temperature.

## Introduction

Oxide semiconductor gas sensors have distinctive advantages, such as high sensitivity, rapid response, simple structure, stability, and reproducibility, and have been widely investigated for combustible gas detection, environmental monitoring, food quality assessment, and exhaled breath analysis^1–9^. However, a simple sensing mechanism based on the charge transfer between the analyte gas and the oxide surface hampers selective gas sensing, and the operation of rigid sensors at elevated temperatures impedes the implementation of wearable chemical sensors^10^.

As a prime example, the selective detection of sub-ppm-level formaldehyde, a probable carcinogen, is critically important for monitoring indoor air quality, but oxide chemiresistors generally exhibit nondiscriminative responses toward a range of interfering indoor pollutants, such as ethanol, carbon monoxide, benzene, toluene, and xylene. In particular, most oxide-based chemiresistors exhibit higher gas responses toward ethanol than formaldehyde as ethanol is reactive and contains a larger amount of C and H for oxidation-based sensing reactions^11,12^. Furthermore, ethanol is ubiquitous, and its typical indoor concentration is often higher than that of formaldehyde, which can cause the formaldehyde sensor to malfunction. Thus, special attention needs to be paid for selective detection of formaldehyde. The loading of various catalysts or the compositional control of sensing materials have been explored but have led to the enhancement of the responses toward ethanol and formaldehyde^13–16^. Furthermore because ethanol and formaldehyde exhibit similarly high reactivity, it is difficult to eliminate only ethanol through oxidative filtering using an catalytic overlayer^17, 18^. In this perspective, the exclusive detection of trace formaldehyde using oxide chemiresistors has been a long-standing challenge.

Molecular sieving or gas separation can be a viable solution when the gases for discrimination are similar in chemistry and/or reactivity but different in molecule size and/or diffusivity^19–24^. Güntner et al.^19^ demonstrated the selective detection of formaldehyde using SnO_2_ sensors placed within a miniaturized chamber that consisted of a gas-separating zeolite/alumina membrane. Tian et al.^20^ and Wang et al.^24^ reported that the coating of a thin ZIF-8 layer on ZnO nanorods enhanced the selectivity to formaldehyde. However, both high selectivity and high response have never been achieved, and further sensor miniaturization, high tunability of molecular sieving, flexible design, and low-temperature operation are required for a wide range of applications.

In this study, a monolithic flexible sensor that can detect ppb-level formaldehyde in a highly selective and sensitive manner at room temperature was designed. The key strategy of sensor design is the two-step screening of analyte gases: the highly selective photoactivation of sensing reactions only toward ethanol and formaldehyde at room temperature and physical filtering of ethanol using a molecular-sieving overlayer. For this, a highly photoactive TiO_2_ sensing film^25^ on a polyethylene terephthalate (PET) substrate was coated with a molecular-sieving ZIF-7/polyether block amide (PEBA) composite overlayer. PEBA with high flexibility, mechanical strength, and compatibility to ZIF-7^26^ enables the coating of thin and homogeneous mixed matrix membrane (MMM) overlayer and offers the flexible sensor design. The TiO_2_ sensor with MMM overlayer exhibited an ultrahigh selectivity (57.4–6754.5 times) for formaldehyde overall indoor pollutants, including ethanol, and a high response (resistance ratio = 2.4 toward 25 ppb formaldehyde) at 23 °C under UV light, whereas the pristine TiO_2_ sensor showed nondiscriminative gas responses. The mechanism underlying high selectivity and response was investigated in relation to the dependence of molecular sieving on the metal organic framework (MOF) structure, the configuration of MMM, and photoactivated gas-sensing reaction. Finally, the potential for flexible formaldehyde sensors is discussed.

## Results

### Fabrication of gas sensors coated with mixed matrix membrane

**Fig. 1:**
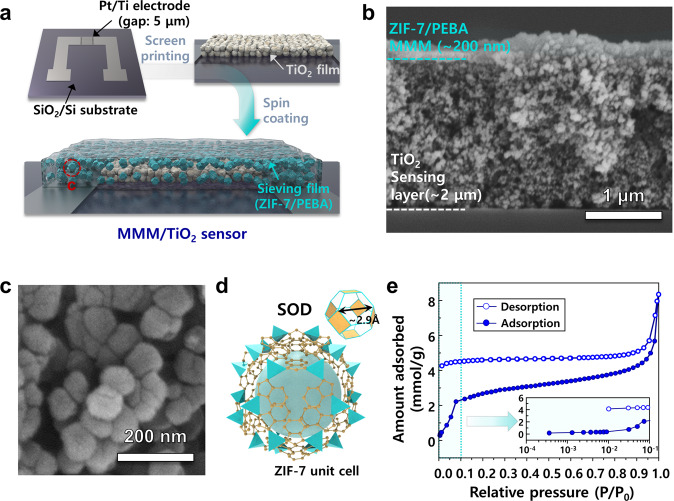
Fabrication and characterization of MMM-coated TiO_2_ sensor. **a** Schematic illustration of steps for the fabrication of MMM(ZIF-7/PEBA) coated TiO_2_ sensors. **b** Cross-sectional SEM images of 5MMM/TiO_2_ sensor, **c** High-magnification SEM image of the ZIF-7, **d** Schematic illustration of ZIF-7 unit cell, **e** N_2_ adsorption/desorption isotherm of ZIF-7 (measured at 77 K).

Figure 1 shows overall design of the monolithic formaldehyde sensor. The sensor is designed in a bilayer structure on a silicon substrate or PET substrate. First, the TiO_2_ slurry was screen printed on a substrate with two Pt/Ti interdigited electrodes (electrode gap 5 μm) and heat treated to fabricate the pristine TiO_2_ sensor. The MMMs (ZIF-7/PEBA composite material) with different contents of ZIF-7 (0, 2.5, 5, 20, and 40 wt% in the final sensing film) were spin coated on the TiO_2_ sensing film (Fig. 1a). For simplicity, hereinafter, these sensors will be referred as ‘*x*MMM/TiO_2_’ (*x* = 2.5, 5, 10, 20, and 40). A cross-sectional view and elemental mapping of the 5MMM/TiO_2_ sensing film (Fig. 1b, Supplementary Fig. 1) showed the coating of an MMM overlayer (thickness: ∼200 nm) on the TiO_2_ sensing film (thickness: ∼2 μm). The size of the primary TiO_2_ particles was ~20 nm. The ZIF-7 nanoparticles used to prepare the MMM overlayer had an average size of 139.2 ± 23.4 nm (Fig. 1c). The XRD pattern of the as-synthesized ZIF-7 nanoparticles was consistent with that of the simulated theoretical pattern (Supplementary Fig. 2). ZIF-7 has a unit cell of a Sodalite (SOD) structure, and the size of the six-membered ring (6MR), the largest pore of the unit cell, is theoretically ~2.9 Å (Fig. 1d)^27^. Therefore, in BET analysis, nitrogen (3.6 Å) is known to be inaccessible to ZIF-7 at 77 K. However, Collados et al.^28^ demonstrated that nitrogen adsorption and desorption occur if the pores of ZIF-7 are activated by the complete removal of DMF remaining from the synthesis. Thus, for the analysis of surface area and pore size distribution, the ZIF-7 nanoparticles were pre-treated at 383 K for 48 h. The desorption and adsorption curves did not meet each other (Fig. 1e). In addition, the adsorption curve increased rapidly near a relative pressure of 1.0 due to the nitrogen condensation at the interparticle space. These results are consistent with those reported by Collados et al.^28^, and the measured total pore volume (0.2072 cm^3^/g) is similar to the theoretical value (0.207 cm^3^/g), confirming the successful synthesis of ZIF-7 with the intended porosity and structure.

The top-view SEM images revealed that the polymer or MMM overlayer was coated uniformly, regardless of the content of ZIF-7 (Supplementary Fig. 3). The ZIF-7 nanoparticles were evenly distributed over the PEBA membrane in the 2.5MMM/TiO_2_ and 5MMM/TiO_2_ specimens (Supplementary Fig. 3b, c), whereas aggregations between the ZIF-7 nanoparticles were frequently observed in the 10MMM/TiO_2_, 20MMM/TiO_2_, and 40MMM/TiO_2_ specimens (Supplementary Fig. 3d–f). This shows that the excessive loading of ZIF-7 nanoparticles is detrimental for dispersing the ZIF-7 nanoparticles within the polymeric matrix.

### Selective detection of ethanol and formaldehyde by photoactivation

The gas-sensing characteristics of pristine TiO_2_, TiO_2_ with polymer overlayer (P/TiO_2_), and *x*MMM/TiO_2_ (*x* = 2.5, 5, 10, 20, and 40) sensors fabricated on SiO_2_ substrates were measured at 23 °C under UV illumination (wavelength: 365 nm, radiation intensity: 225 mW, distance between the sensor and the light source: 1 cm). All sensors exhibited typical n-type oxide semiconductor behavior. That is, the resistance decreased upon exposure to a reducing gas and then recovered to its original value in air (Supplementary Fig. 4). Thus, the gas response (*S*) was defined as (*R*_a_ − *R*_g_)/*R*_g_, where *R*_a_ and *R*_g_ are the resistances in air and gas, respectively.

**Fig. 2:**
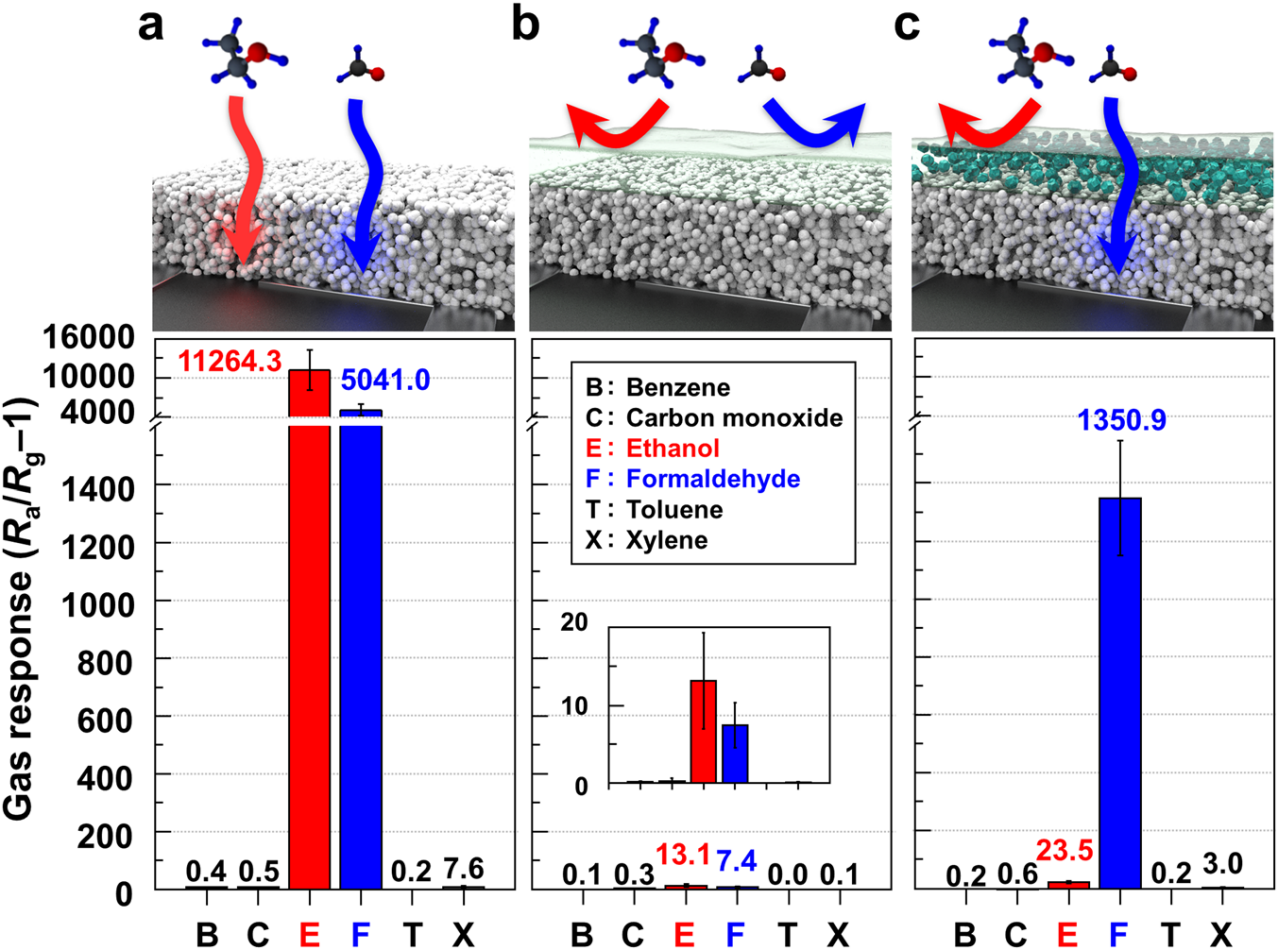
Tailoring of gas selectivity. **a** Gas responses of a bare TiO_2_, **b** Pure PEBA/TiO_2_, and **c** 5MMM/TiO_2_ sensors exposed to 5 ppm benzene (B), carbon dioxide (C), ethanol (E), formaldehyde (F), toluene (T), and p-xylene (X) at 23 °C under UV illumination (wavelength: 365 nm). Error bars represent s.d. of the mean.

The pristine TiO_2_ sensor exhibited a high response to formaldehyde (*S* = 5041.0), but the response to ethanol was even higher (*S* = 11264.3), making the discrimination between the two gases difficult (Fig. 2a). Interestingly, responses to CO, benzene, toluene, and xylene were negligibly low (0.2–7.6). To understand this, the sensing characteristics of the TiO_2_ sensor were measured at elevated temperatures (300–375 °C) in the absence of UV light (Supplementary Fig. 5). The responses to 6 different gases were relatively low (*S* = 0.7–18.1) and the exclusive detection of a specific gas was difficult because the responses to most gases except CO were not negligible and comparable. Furthermore, the sensor showed the highest response to ethanol, and no formaldehyde selectivity was observed.

This suggests that the photoactivation of the TiO_2_ sensor at room temperature, unlike thermally activated gas sensing at high temperature, not only significantly promotes the sensing of ethanol and formaldehyde but also completely eliminates the cross responses to other interference gases, enabling selective sensing of ethanol and formaldehyde. This is in line with the gas-sensing results of UV-assisted TiO_2_ sensors in the literature^29^.

Even considering high reactivity, the responses of pristine TiO_2_ sensor to ethanol and formaldehyde at 23 °C under UV light are very high. In order to understand the role of photo-assisted activation in the gas-sensing reaction, I–V characteristics of the TiO_2_ sensor under dark conditions and UV light were compared (Supplementary Fig. 6). The sensor showed negligibly low current (e.g., 1.6 pA at 1 V) under dark conditions, which made the measurement of gas-sensing characteristics difficult. Under UV light illumination, however, the current at 1 V increased to 1398.8 pA and the sensor resistance became measurable. The 874-fold increase in current after UV light illumination clearly demonstrates the photo-generation of charge carriers in the TiO_2_ sensor.

The P25 TiO_2_ fine powders used in this study were a mixture of rutile and anatase phases, which are reported to exhibit superior photocatalytic properties due to the elongated lifetimes of photo-excited charge carriers by the charge-funneling energy band alignment at the rutile-anatase interface^30^. Interestingly, the pristine TiO_2_ sensors with a single phase (rutile or anatase) showed relatively low gas responses even under UV illumination (Supplementary Fig. 7), verifying that the photo-excitation and charge separation in the mixed phase of TiO_2_ played a key role in promoting the gas-sensing reaction. This is supported by the fact that the incident photon to electron efficiency of mixed TiO_2_ phase under the UV light (wavelength = 365 nm) is substantially higher than those of rutile and anatase phases (Supplementary Fig. 8). When TiO_2_ is in a dark state, chemically adsorbed oxygen ions (O_2_^−^_(chem-ads)_) at room temperature are relatively stable and it is difficult to remove O_2_^−^_(chem-ads)_ with large adsorption energy from the TiO_2_ surface (Supplementary Fig. 9a, b). However, under UV light, photo-induced electrons react with ambient oxygen molecules to adsorb more oxygen ions (O_2_^−^_(photo-ads)_) and photo-induced holes react with O_2_^−^_(photo-ads)_, making their desorption easier (Supplementary Fig. 9c). Unlike chemically adsorbed oxygen ions, oxygen ions adsorbed through photoreaction are relatively weakly bound to the surface of TiO_2_, so they can easily react with reducing gases, leading to high chemiresistive variations (Supplementary Fig. 9d). The optical bandgap of the P25 TiO_2_ used in this study is known to be 3.1 eV, so UV with a wavelength of 365 nm is sufficient to activate TiO_2_. Accordingly, the high responses to ethanol and formaldehyde in Fig. 2a are explained by the photoactivated sensing of highly reactive gases.

The chemiresistive variation in oxide semiconductors depends on the change in the charge density caused by the reaction between the gas and the adsorbed oxygen. If the same concentration of different gases with similar reactivity are detected, a gas with a higher molecular weight reacts with more adsorbed oxygen, resulting in a higher gas response. Formaldehyde is a by-product gas that can be formed during the oxidation of ethanol and has a relatively low molecular weight^31^. Accordingly, in the literature, the response to formaldehyde is lower than that to ethanol^11, 12^. Furthermore, it is challenging to find catalysts for the highly selective promotion of the formaldehyde sensing reaction or to design a catalytic filtering layer to oxidize only ethanol without oxidizing formaldehyde. Indeed, the catalytic filtering layer formed on the top of the sensing film decreased both responses to ethanol and formaldehyde^17, 18^. This means that the exclusive detection of formaldehyde via catalyst loading or by using a catalytic filtering layer is highly challenging even in photo-assisted gas sensing at room temperature, which requires a new strategy such as molecular sieving.

In the P/TiO_2_ sensor, the responses to ethanol and formaldehyde significantly decreased to 13.1 and 7.4 (Fig. 2b), respectively, which correspond to 1/860 and 1/681 of those in the pristine TiO_2_ sensor, respectively. Responses to other gases ranged from 0 to 0.3. This implies that most of the gases were blocked by the relatively dense PEBA membrane, and only trace amounts of gas permeated. When gas permeates through a pure polymer membrane, such as PEBA, it dissolves into the polymer and then diffuses. Thus, the low responses to ethanol and formaldehyde can be attributed to the trace amount of gas transport across the polymer membrane through dissolution and diffusion. However, this cannot be used to improve gas selectivity because of the lack of a selective molecular-sieving effect. Furthermore, the amount of gas permeation is too small to achieve a high response.

### Exclusive detection of formaldehyde by combining molecular sieving and photoactivation

Interestingly, the 5MMM/TiO_2_ sensor exhibited a very high response (*S*_F_ = 1350.9) to 5 ppm formaldehyde, but its ethanol response became negligible (*S*_E_ = 23.5) (Fig. 2c). That is, the coating of the 5MMM layer on the TiO_2_ sensor slightly decreased the formaldehyde response but dramatically eliminated the ethanol response. Thus, the formaldehyde response became 57.4 times higher than the ethanol response and 450.3–6,754.5 times higher than that of the other four interference gases, demonstrating the exclusive detection of formaldehyde.

The six-membered ring (6MR) pore size of ZIF-7 was calculated to be ~0.3 nm, which is slightly smaller than the kinetic diameter of formaldehyde (0.373 nm). However, the BET results in Fig. 1e reveal that N_2_ with a kinetic diameter of 0.36 nm, which is larger than that of 6MR pores, reached the inner pores of ZIF-7. This is also in line with other reports on gas separation using ZIF-7. For instance, Gücüyener et al.^32^ reported that carbon-containing compounds larger than the theoretical pore size of ZIF-7 can enter the pores of ZIF-7, leading to the separation of C_2_H_4_ (0.41 nm) and C_2_H_6_ (0.44 nm). Bergh et al.^33^ also reported that ZIF-7 can be utilized to separate C_3_H_8_ (0.43 nm) and C_3_H_6_ (0.46 nm). These results suggest that the threshold size of gas molecules for separation is slightly larger than the theoretical value (0.3 nm). From this perspective, ZIF-7 nanoparticles can be expected to separate formaldehyde (0.373 nm) from larger interfering indoor pollutants, such as ethanol (0.45 nm), toluene (0.585 nm), benzene (0.585 nm), and xylene (0.585 nm) (Supplementary Fig. 10). For further investigation, 5MMM layer was coated on quartz support and the outlet gas after filtering the gas mixture of 1 ppm formaldehyde and 1 ppm ethanol was analyzed using proton transfer reaction quadruple mass spectroscopy (Supplementary Fig. 11). The result showed the sieving of formaldehyde with ethanol filtering, confirming the molecular-sieving effect. The negligibly low response to CO, which has a small size (0.376 nm), can be attributed to the lower reactivity, which is supported by the lowest CO response of the pristine TiO_2_ sensor even at elevated temperatures (Supplementary Fig. 5). Thus, the exclusive detection of formaldehyde using the 5MMM/TiO_2_ sensor in the present study is explained by the synergistic combination of the molecular sieving of gas using MMMs containing ZIF-7 and the highly selective photoactivation of gas-sensing reactions toward reactive formaldehyde and ethanol at room temperature. It should be pointed out that such a molecular-sieving effect cannot be achieved when cracks exist in the MMM layer. That is, in the present study, the ZIF-7 particles were well-dispersed in 5MMM without any cracks, and the 5MMM overlayer was uniformly coated on the TiO_2_ film without defects, thereby allowing molecular sieving without deterioration of mass transport.

**Fig. 3:**
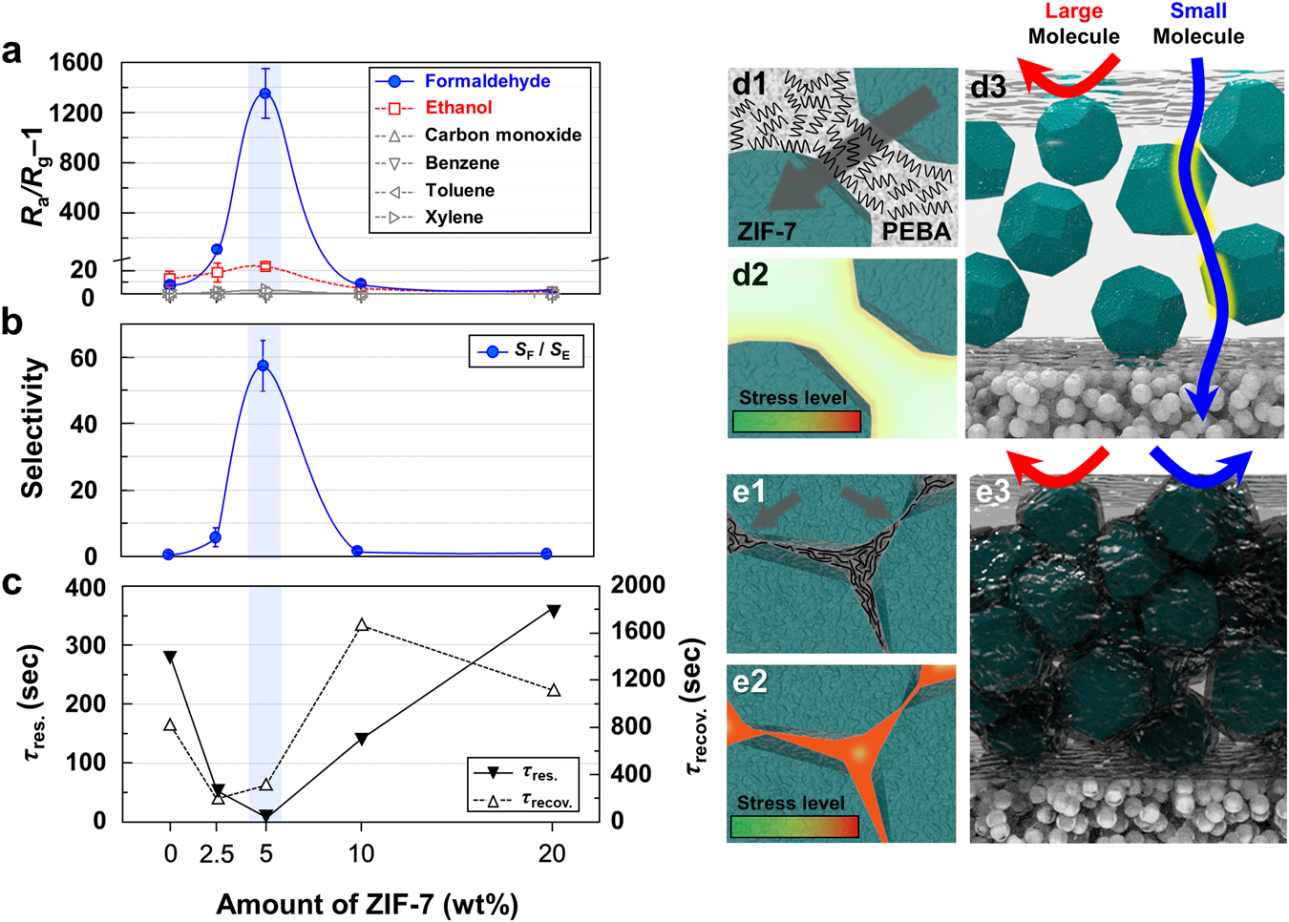
Gas response, formaldehyde selectivity, response/recovery times, and gas-sensing mechanism. **a** Gas response of pure PEBA/TiO_2_, 2.5MMM/TiO_2_, 5MMM/TiO_2_, 10MMM/TiO_2_, 20MMM/TiO_2_ sensors (temperature: 23 °C). **b** Formaldehyde selectivity to ethanol of pure PEBA/TiO_2_, 2.5MMM/TiO_2_, 5MMM/TiO_2_, 10MMM/TiO_2_, 20MMM/TiO_2_ sensors. Error bars represent s.d. of the mean. **c** 90% response time (τ_res._, left) and 90% recovery times (τ_recov._, right) of pure PEBA/TiO_2_, 2.5MMM/TiO_2_, 5MMM/TiO_2_, 10MMM/TiO_2_, and 20MMM/TiO_2_ sensors. **d, e** Schematic illustration of gas penetration model, stress level, and polymer configuration when mild amount of ZIF-7 loading in PEBA (**d**) and excessively high amount of ZIF-7 loading in PEBA (**e**).

The contents of ZIF-7 in MMM coated on TiO_2_ sensor were changed from 2.5 to 20 wt% and their gas-sensing characteristics were measured (Fig. 3a). In all sensors, the responses to formaldehyde and ethanol were the highest. Thus, the selectivity to formaldehyde over ethanol interference (*S*_F_/*S*_E_) was calculated (Fig. 3b). As the loading concentration of ZIF-7 increased to 2.5 wt%, the responses to formaldehyde and ethanol increased to 107.2 and 18.3, respectively. Compared to those of the P/TiO_2_ sensor, the gas responses increased up to 14.5 times, indicating that more analyte gas passed through the porous ZIF-7 nanoparticles dispersed in the 2.5MMM overlayer. The *S*_F_/*S*_E_ value of the 2.5MMM/TiO_2_ sensor was 5.9, which was approximately 9.8 times higher than that (*S*_F_/*S*_E_ = 0.6) of the P/TiO_2_ sensor, clearly demonstrating that the permeation of formaldehyde through 2.5MMM is greater than that of ethanol, which suggests the contribution of ZIF-7 nanoparticles for moderate molecular sieving. The further enhancement of the *S*_F_ value with increasing ZIF-7 content to 5 wt% can be attributed to the increase in mass transport through the 5MMM overlayer. It is worth noting that high *S*_F_/*S*_E_ values (57.4) primarily occurred owing to molecular sieving. However, excessive loading of ZIF-7 (10–20 wt%) on MMM deteriorated both the response and selectivity to formaldehyde. For instance, compared to the 5MMM/TiO_2_ sensor, the 10MMM/TiO_2_ sensor showed a very low selectivity (*S*_F_/*S*_E_ = 1.8) with 1/150 times lower formaldehyde response (*S*_F_ = 9.0) and 1/4.7 times lower ethanol response (*S*_E_ = 5.0). Furthermore, 20MMM/TiO_2_ showed formaldehyde (*S*_F_ = 1.1) and ethanol (*S*_E_ = 1.4) responses even lower than those of P/TiO_2_ and did not show notable responses to most gases.

This should be examined under the frameworks of the amount and dispersion of inorganic particles in the polymer matrix and the configuration of organic–inorganic interfaces^34–36^. When only the polymer is present, the volume of the polymer decreases as the solvent evaporates, and the polymer chains maintain a random configuration that enables the short-distance gas transport. However, the polymeric film in P/TiO_2_ sensor is too thick for the transport over long distance, leading to low and comparable responses to ethanol and formaldehyde as shown in Fig. 2b. Accordingly, the thick and pure polymer overlayer does provide neither sufficient gas transport nor selective gas sieving. When hard inorganic particles (e.g., ZIF-7) are dispersed in the polymer, the polymer molecules around the inorganic particles affix to the surface of the inorganic particles^34^. Because the contraction of the polymer near the particles is significantly inhibited, the polymer stretches over the particle surface, creating compressive stress. In the present study, when the amount of ZIF-7 nanoparticles is not excessively high, the compressive stress developed near the surface of inorganic particles could be effectively released by the adjacent polymer layer (Fig. 3d1,d2). Under this condition, the introduction of porous ZIF-7 fillers decreases the interfiller spacing, which facilitates the gas transport across thin polymeric layer between fillers as well as molecular sieving via ZIF-7 (Fig. 3d3). Accordingly, the coating of 2.5MMM or 5MMM layers significantly enhanced not only the gas response but also the gas selectivity to formaldehyde. However, if the amount of ZIF-7 nanoparticles becomes excessively high, a large number of compressive stress fields are generated and the concentration of stress fields over the entire matrix significantly rigidifies the polymer (Fig. 3e1,e2). Under such an overlapped stress field, the polymer chains are arranged not in a random-coil-like structure but in a stacked form centering around ZIF-7, so that the secondary bonds between the chains increase, the mobility of the chains is greatly limited, and rigidifying occurs^35, 36^. Accordingly, the polymer near the surface of the ZIF-7 nanoparticles becomes impermeable, thereby restricting mass transport and disabling molecular sieving through the ZIF-7 nanoparticles (Fig. 3e3). The low response and selectivity to formaldehyde in the 10MMM/TiO_2_ and 20MMM/TiO_2_ sensors can be understood from this viewpoint. This tendency can be confirmed by examining the glass transition temperature (T_g_). In general, the interfacial interaction and the concentration of stress fields weaken the mobility of the polymer chain, leading to an increase in T_g_^37, 38^. The T_g_ values of membranes were determined based on the midpoint temperature of the transition in the Differential Scanning Calorimeter (DSC) (temperature: −70–50 °C, heating rate: 10 °C/min) curve. The T_g_ of pristine PEBA was −47.4 °C, and the T_g_ values of the MMM layers gradually increased to −44.3 °C as the amount of ZIF-7 increased to 20 wt% (Supplementary Fig. 12), which supports the development and accumulation of stress at the particle-polymer interfaces. The mass transport of analyte gases across the MMM layers can also be examined using the response/recovery time of the sensor. For this, the 90% response and recovery times (τ_res_ and τ_recov_), that is, the time required to reach 90% resistance variation upon exposure to 5 ppm formaldehyde and air, respectively, were calculated from the sensing transients (Fig. 3c). When 2.5 and 5 wt% of ZIF-7 particles were loaded, both the τ_res_ and τ_recov_ values decreased significantly compared to those of the P/TiO_2_ sensor, suggesting the enhancement of mass transport by the introduction of ZIF-7 nanoparticles. In particular, the τ_res_ value of the 5MMM/TiO_2_ sensor was as short as 9 s, demonstrating the potential for the real-time monitoring of formaldehyde. In contrast, when the loading amount of ZIF-7 increased from 10 to 20 wt%, the τ_res_ and τ_recov_ values increased significantly, indicating decreased gas permeation due to the rigidity of the polymer. It is worth noting that the dramatic decreases of gas response and responding speed with increasing the amount of ZIF-7 from 5 wt% to 10–20 wt% exclude the possibility to generate nonselective voids between polymer and ZIF-7 filler.

The TiO_2_ sensor coated with thicker 5MMM overlayer (thickness: 600 nm) exhibited the lower response to formaldehyde (*S*_F_ = 292.8) but higher selectivity (*S*_F_/*S*_E_ = 139.5), indicating that the change of overlayer thickness provides further control over gas-sensing characteristics (Supplementary Fig. 13). To understand the role of MMM structures in gas filtering, a ZIF-7 overlayer without a polymeric component was coated on the TiO_2_ film. The cohesion between the TiO_2_ sensing film and ZIF-7 was poor and the TiO_2_ sensing layer was chemically affected during the formation of the overlayer. In contrast, the bonding between MMM and the TiO_2_ layer in the present study was strong and stable. This clearly shows that a bilayer with a molecular-sieving MMM overlayer is a cost-effective and versatile approach to design a formaldehyde sensor with excellent selectivity and sensitivity.

### Low detection limit, selectivity, dynamic response, and stability

**Fig. 4:**
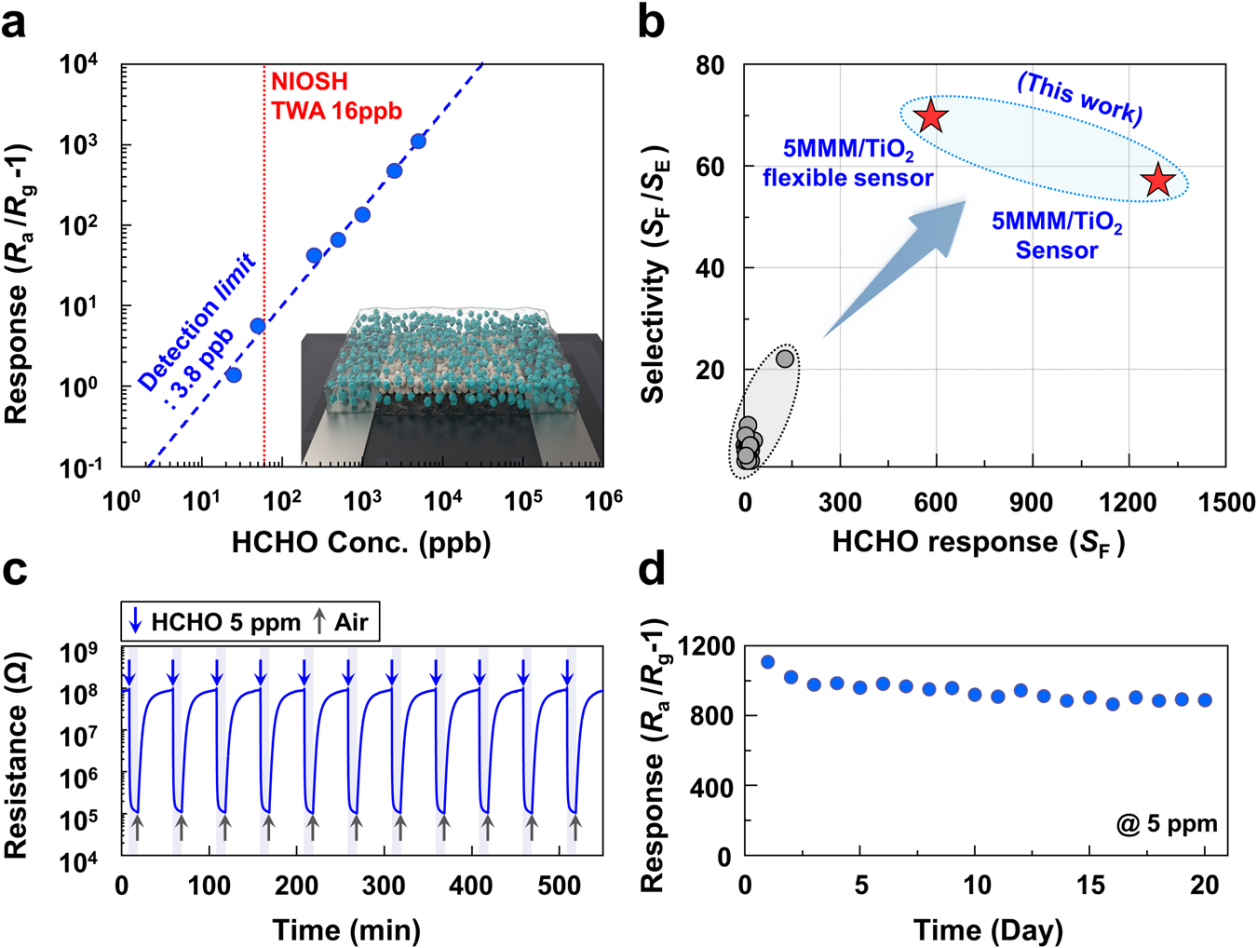
Low detection limit, formaldehyde selectivity/response, dynamic response, and stability. **a** Gas response as function of formaldehyde concentration. **b** Formaldehyde selectivity (*S*_F_/*S*_E_) and response (*S*_F_) compared to the reported values in the literature. **c** Repeated sensing transients to 5 ppm formaldehyde at 23 °C under 365 nm UV radiation. **d** Long-term stability of 5MMM/TiO_2_ sensor (UV light illumination during sensor measurement) S1-S22.

Many attempts have been made to improve the sensing properties of formaldehyde. However, most sensors did not measure the selectivity to ethanol, and even if this was measured, both high selectivity and high response to formaldehyde have never been accomplished at the same time. The sensing transients of 0.025–5 ppm formaldehyde of the 5MMM/TiO_2_ sensor were measured (Supplementary Fig. 14). From the concentration-response relationship in Fig. 4a, the detection limit of formaldehyde was evaluated to be as low as 0.0038 ppm when the criterion of *R*_a_/*R*_g_ − 1 > 0.2 was used for gas sensing, which is far lower than the 8 h-weighted average permissible exposure limit (0.016 ppm) in the workplace as well as the ceiling limit of exposure (0.1 ppm) established by The National Institute for Occupational Safety and Health^39^. This demonstrates that the present sensor provides a promising solution for the exclusive detection of ppb-level formaldehyde for monitoring indoor air quality. It is worth noting that both the formaldehyde response and selectivity of 5MMM/TiO_2_ are the highest among all monolithic formaldehyde sensors reported in the literature (Fig. 4b, Supplementary Table 1). The sensor exhibited highly reproducible sensing and recovery upon repeated exposure to 5 ppm formaldehyde (Fig. 4c), similar sensing characteristics at 15–50 °C (Supplementary Fig. 15), and good long-term stability over 20 days (Fig. 4d). The gas-sensing transients to the mixture of formaldehyde (0.5–2.5 ppm) and ethanol (2.5 ppm) were nearly the same to those to formaldehyde (0.5–2.5 ppm) (Supplementary Fig. 16), confirming the successful sensor operation in the mixed gas environment. Similar to most oxide chemiresistors, the ambient moisture deteriorated the gas responses (Supplementary Fig. 17a), suggesting that further investigation is required to design the sensor with endurance against moisture. No notable change in gas-sensing characteristics was found after annealing the sensor at 80 °C (Supplementary Fig. 18), which can be used to refresh the sensor by mild heat treatment. To check the possibility to design humidity independent gas sensors with molecular-sieving layer, WO_3_ sensor with 5MMM overlayer has been examined. The coating of 5MMM overlayer enabled the ultrahigh selectivity to formaldehyde (Supplementary Fig. 17b, c), which reconfirms the general validity of molecular sieving. Furthermore, the sensor maintained a high formaldehyde selectivity regardless of humidity variation (Supplementary Fig. 17b), demonstrating that the combination between diverse oxide chemiresistors and MMM overlayer can provide a solution to mitigate water poisoning. The amount of water vapor adsorption in 5MMM under 80% relative humidity at 25 °C determined by dynamic vapor sorption was 11.5% (Supplementary Fig. 19), which suggests that the 5MMM overlayer can be used in usual ambient atmosphere.

### Flexible formaldehyde sensor

**Fig. 5:**
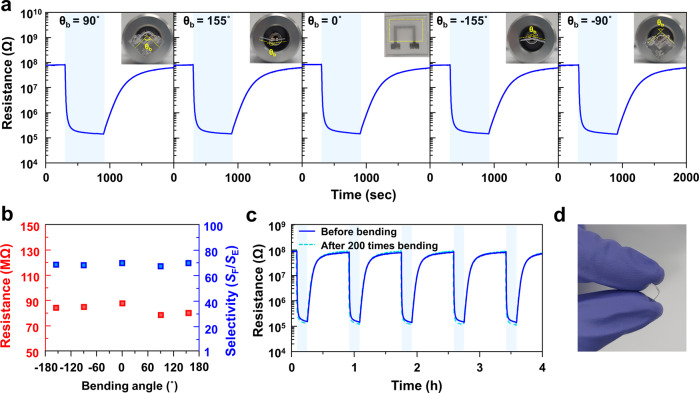
Formaldehyde sensing characteristics of flexible sensor. **a** Gas-sensing transients of 5MMM/TiO_2_/PET sensor to 5 ppm formaldehyde under various bending conditions (temperature: 23 °C). **b** Baseline resistance(left) and formaldehyde selectivity to ethanol(right) of 5MMM/TiO_2_/PET sensor to 5 ppm formaldehyde and ethanol at different bending angles. **c** Repeated sensing transient of 5MMM/TiO_2_/PET sensor before and after 200 bending cycles. **d** Flexibility of a 5MMM/TiO_2_/PET sensor.

In order to examine the potential application of the present sensor design in flexible devices, a flexible formaldehyde sensor was fabricated by coating a TiO_2_ sensor on a PET substrate with a 5MMM overlayer. The response characteristics to formaldehyde and ethanol were investigated in the flat sensor configuration at four different bending angles (−155°, −90°, 90°, and 155°) (Fig. 5a). The flexible 5MMM/TiO_2_/PET sensor showed a very high response to 5 ppm formaldehyde (*S*_F_ > 570) and high selectivity over ethanol interference (*S*_F_/*S*_E_ = 69.9). Furthermore, the bending of sensors barely influenced the sensor resistance, gas response, and selectivity (Fig. 5a, b). Finally, the sensing transient to 5 ppm formaldehyde remained unchanged after the sensor underwent 200 bending cycles (Fig. 5c). The robustness of sensor against repeated bending can be attributed to the unique design of the present sensor, that is, the TiO_2_ sensing layer sandwiched between two polymeric layers (PET and MMM). The formaldehyde sensing behavior in the present study emanated from the synergistic combination between the highly permeable and uniform molecular-sieving overlayer using a unique MOF-polymer composite membrane and UV light-enhanced selective gas-sensing reaction at room temperature. Furthermore, a flexible gas sensor can be designed, and the use of a flexible substrate and MMM layer promises the robustness of the sensor against repeated bending. It is worth noting that recent progress on the light-activated gas sensors enables a monolithic design of photoactivated oxide sensor in contact with micro-LED (size: 30 × 30 μm^2^) with low power consumption (0.184 mW)^40^. Furthermore, flexible design of LED is also available^41^. In this perspective, the ultrahigh selectivity, high response, highly miniaturized monolithic sensor design, room-temperature operation, and flexible design of sensor by molecular sieving and photoactivation can open new pathways for the design of various new gas sensors, including high-performance formaldehyde sensors for accurate indoor monitoring.

## Discussion

In the present study, we designed a new monolithic and flexible sensor that can exclusively detect ppb-level formaldehyde at room temperature without the interference of other indoor air pollutants. For this, TiO_2_ sensing film coated with MMM overlayer composed of a molecular-sieving zeolitic imidazolate framework ZIF-7 and polymer has been used under UV illumination. The photoactivation of gas-sensing reactions at room temperature provided a distinctive pathway to achieve ultrahigh selectivity toward reactive gases such as ethanol and formaldehyde, whereas the thermal activation of sensor led to nonselective gas-sensing characteristics. The MMM overlayer has been adopted to further improve the gas selectivity. The low content of MOFs in MMM limited gas transport and molecular sieving, whereas excessively high MOFs concentrated the stress at the MOF-polymer interface, restricting gas transport. In contrast, a moderate amount of ZIF-7 particles well-dispersed within the polymer matrix facilitated both gas transport and molecular sieving, leading to the high selectivity and response to formaldehyde.

The ultraselective detection of carcinogenic formaldehyde using oxide chemiresistors without the interference of a ubiquitous and reactive ethanol has been a long-standing challenge. In the present study, the synergistic combination of room-temperature photoactivation and molecular sieving, two distinctive approaches to enhance gas selectivity, has been suggested as a new strategy to design exclusive gas sensors. Furthermore, a unique sensor design, a TiO_2_ film sandwiched between two flexible layers, a lower polyethylene terephthalate substrate and upper MMM layer, exhibited high selectivity, high response, room-temperature operation, flexibility, monolithic design, and high reversibility, which can open new pathways for indoor air monitoring and flexible electronics.

## Methods

**Preparation of ZIF-7 nanoparticles.** Benzimidazole (20 mmol, 1,3-Benzodiazole, ≥99.0%, Sigma–Aldrich, USA) was added to 100 mL of methanol. A separate solution was prepared by dissolving 5 mmol of zinc nitrate hexahydrate (Zn(NO_3_)_2_·6H_2_O, 98%, Sigma–Aldrich, USA) in 50 mL of N,N-dimethylformamide (HCON(CH_3_)_2_, 99.9%, Sigma–Aldrich, USA). The Zn salt solution was poured into the benzimidazole solution and stirred for 24 h at room temperature. The resulting suspension was centrifuged at 11,984 × *g* for 10 min, and the precipitate was washed three times with methanol. The product was vacuum-dried at 120 °C for 24 h for characterization. When the ZIF-7 nanocrystals were used as the fillers for MMMs, the as-synthesized nanocrystals were dispersed in an excess amount of fresh methanol for 48 h to completely remove the residual DMF. Subsequently, the nanocrystals were centrifuged from methanol and re-dispersed into a mixture of deionized (DI) water/ethanol (30/70 wt%) to prevent particle agglomeration.

**Preparation of TiO**_2_** sensing film.** The slurry for the sensing film was prepared by mixing titanium (IV) oxide powders (P25, TiO_2_, ≥99.5%, Aeroxide® TiO_2_, Germany) with a terpineol-based ink (FCM, USA) at a ratio of 1:6 (by weight). The sensing film was screen printed on silicon oxide (SiO_2_) substrates (area: 1.0 × 1.0 mm^2^; thickness: 0.68 mm) with two interdigitated Pt electrodes (IDE) on the upper surface (electrode gap: 5 μm). After screen printing, the sensing film was heat treated at 450 °C for 2 h to remove organic components. In order to prepare the flexible gas sensor, the same TiO_2_ slurry was screen printed on the IDE-patterned PET substrate (area: 0.8 × 0.8 mm^2^; thickness: 0.34 mm). Then, the organic components were removed by heat treatment at 130 °C overnight. The TiO_2_ sensing films were ~2 μm thick.

**Preparation of gas-filtering membrane overlayer.** Pebax®1657 granules (Arkema, France) containing 40 wt% polyamide and 60 wt% polyethylene glycol were mixed with H_2_O/ethanol (30/70 wt%) and the solution was vigorously stirred at 80 °C for 2 h. The amount of Pebax®1657 was fixed at 2 wt%. The Pebax®1657 solution and the ZIF-7 suspension were mixed directly without any pretreatment, and then sonicated for 10 min, followed by stirring for 24 h at room temperature. Before coating the overlayer, this slurry was sonicated to remove any bubbles. The ZIF particles dispersed well in suspension, and thus no precipitate was observed during the mixing process. The filler (ZIF) loading was calculated as follows:

$${{{{{\rm{Particle}}}}}}\,{{{{{\rm{loading}}}}}}\,({{{{{\rm{wt}}}}}}\, \% )={{{{{\rm{weight}}}}}}\,{{{{{\rm{of}}}}}}\,{{{{{\rm{particles}}}}}}/({{{{{\rm{weight}}}}}}\,{{{{{\rm{of}}}}}}\,{{{{{\rm{particles}}}}}}\;+\;{{{{{\rm{weight}}}}}}\,{{{{{\rm{of}}}}}}\,{{{{{\rm{polymer}}}}}})\times 100$$

The gas-filtering overlayer was coated on the TiO_2_ sensing film through two-step spinning (1st spinning 1000 rpm for 3 s; 2nd spinning 1500 rpm for 90 s) of the above suspension. After spin coating, the films were dried at 70 °C for 24 h. Experimental details for material characterization and measuring gas-sensing characteristics are given in the supporting information.

## Supplementary information

Supplementary Information

## Data Availability

The data that support the findings of this study are available from the corresponding author upon reasonable request.
