# Supplementary material for: Exclusive and ultrasensitive detection of formaldehyde at room temperature using a flexible and monolithic chemiresistive sensor
Source: Nat Commun. 2021 Aug 16;12:4955. doi: 10.1038/s41467-021-25290-3 (PMC8368006; doi:10.1038/s41467-021-25290-3)
Supplement: Supplementary file 1 — Supplementary Information [file 41467_2021_25290_MOESM1_ESM.pdf]

Supplementary Information for

Exclusive and Ultrasensitive Detection of Formaldehyde at Room Temperature using a Flexible and Monolithic Chemiresistive Sensor

Yong Kun Jo[1], Seong-Yong Jeong[1], Young Kook Moon[1], Young-Moo Jo[1], Ji-Wook Yoon[2], and Jong-Heun Lee[1]*

[1]Department of Materials Science and Engineering, Korea University, Seoul 02841, Republic of Korea

[2]Department of Information Materials Engineering, Jeonbuk National University, Jeonju 54896, Republic of Korea

*e-mail: jongheun@korea.ac.kr

Supplementary Methods

Characterization of materials

FE-SEM (SU-70, Hitachi Co. Ltd., Japan) was used to investigate the morphologies and microstructures of the materials and sensing films. FE-SEM-EDS (AURIGA, Carl Zeiss, Germany) was used to obtain elemental mapping of monolithic sensing films. The pore-size distribution and surface area of ZIF-7 were analyzed using the Brunauer–Emmett–Teller (BET, ASAP2020, Micromeritics, USA) method. The crystal structure was characterized using X-ray diffraction (XRD, D/MAX-2500 V/PC, Rigaku, Japan) with a CuKα radiation source (λ = 1.5418 Å). The thermal properties and glass transition temperatures (T_g) of the membranes were measured with a DSC thermal analyzer (DSC214 Polyma, NETZSCH, Germany) at a heating rate of 10 °C/min in the temperature range of -90−200 °C under a nitrogen atmosphere. The water vapor sorption of the membrane was measured with a dynamic vapor sorption (DVS intrinsic-1, Surface Measurement Systems Ltd., United Kingdom)

Gas-sensing characteristics

The sensor was placed in a specially designed quartz cube (inner volume: 1.5 cm^3), and the atmosphere was controlled via an automatic four-way valve. The flow rate of the gas was fixed at 200 cm^3 min^{-1}. An electrometer (Picoammeter 6487, Keithley, Tecktronic Inc., USA) interfaced with a computer was used to measure the direct-current two-probe resistance. UV LED lamp (wavelength 365 nm; voltage: 3.5 V; maximum power: 1 W, maximum current: 350 mA) was attached to the outside of quart cube (gas sensing chamber) to activate sensor. The distance between the sensor and light source was 10 mm.

Preparation of WO_3 sensing film.

The slurry for the sensing film was prepared by mixing Tungsten (VI) oxide powders (powder, ≤25 μm, ≥99% trace metals basis, sigma-aldrich, USA) with a terpineol-based ink (FCM, USA) at a ratio of 1:6 (by weight). The sensing film was screen-printed on silicon oxide (SiO_2) substrates (area: 1.0 × 1.0 mm^2; thickness: 0.68 mm) with two interdigitated Pt electrodes (IDE) on the uppe surface (electrode gap: 5 μm). After screen printing, the sensing film was heat-treated at 450 °C for 2 h to remove organic components.

Gas permeation test

The permeation test of the 5MMM layer were evaluated in a quartz tube reactor (length: 400 mm, inner diameter: 8 mm). 5MMM layer (thickness: 1 mm) was loaded onto the porous quartz support placed in the middle of the quartz tube. The total flow rate of the reactant gases (1 ppm ethanol + 1 ppm formaldehyde, N_2 base) was 45 cm^3 min^{-1}. The permeate stream were analyzed by on-line PTR-QMS (PTR-QMS 300, Ionicon Analytik, Austria). The drift tube conditions were fixed (voltage: 600 V, temperature: 80 °C, pressure: 2.3 mbar) and the electric field strength/gas number density (E/N) was 136 Td (1 Td = 10^{-17} V cm^2). The H_3O^+ ions served as a primary ion.

Dynamic vapor sorption (DVS) test

The Dynamic Vapor Sorption (DVS) technique automatically generates the different humidity levels for measuring the sorption isotherm. To determine the time when equilibrium is reached the instrument records the change in mass over time (dm/dt). When dm/dt is below 0.002% the next humidity level is automatically set.

Sample prepared for DVS analysis were prepared by drop-coating 5MMM solution (5wt% ZIF-7/PEBA, 5ml) on a 6-inch silicon wafer, dried at 70 °C for 24 h, and peeled off. The initial weight of the sample used for DVS analysis was 9.51 mg . As testing conditions, an air temperature of 25 °C and a range of relative air humidity from 0–95% were chosen. The steps of humidity were set to around 5% and the mass changes were recorded (interval time: 20 s). Anytime the control program detected a change in mass smaller than 0.002% per 10 minute, the relative humidity changed automatically by around 5%. The measurement of the sorption isotherm was complete after about 22 h.

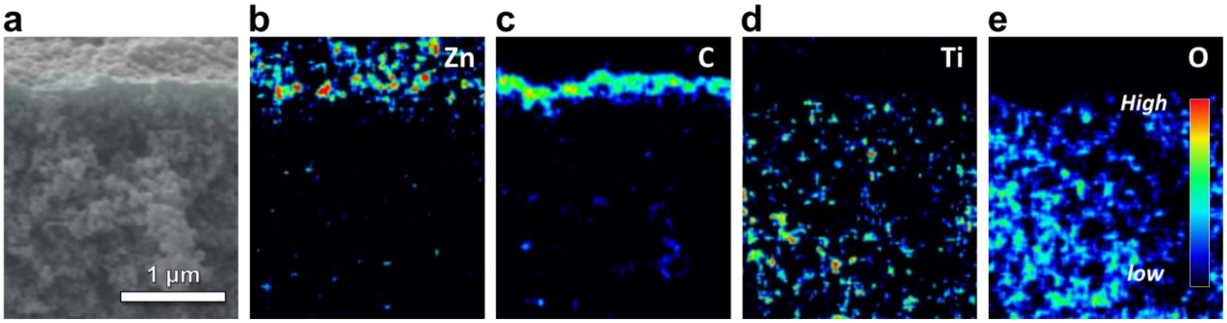

Supplementary Fig. 1 a Cross-sectional SEM image of 5MMM/TiO_2 **b-e** EDS elemental (Zn, C, Ti, and O) mapping of 5MMM/TiO_2 sensor.

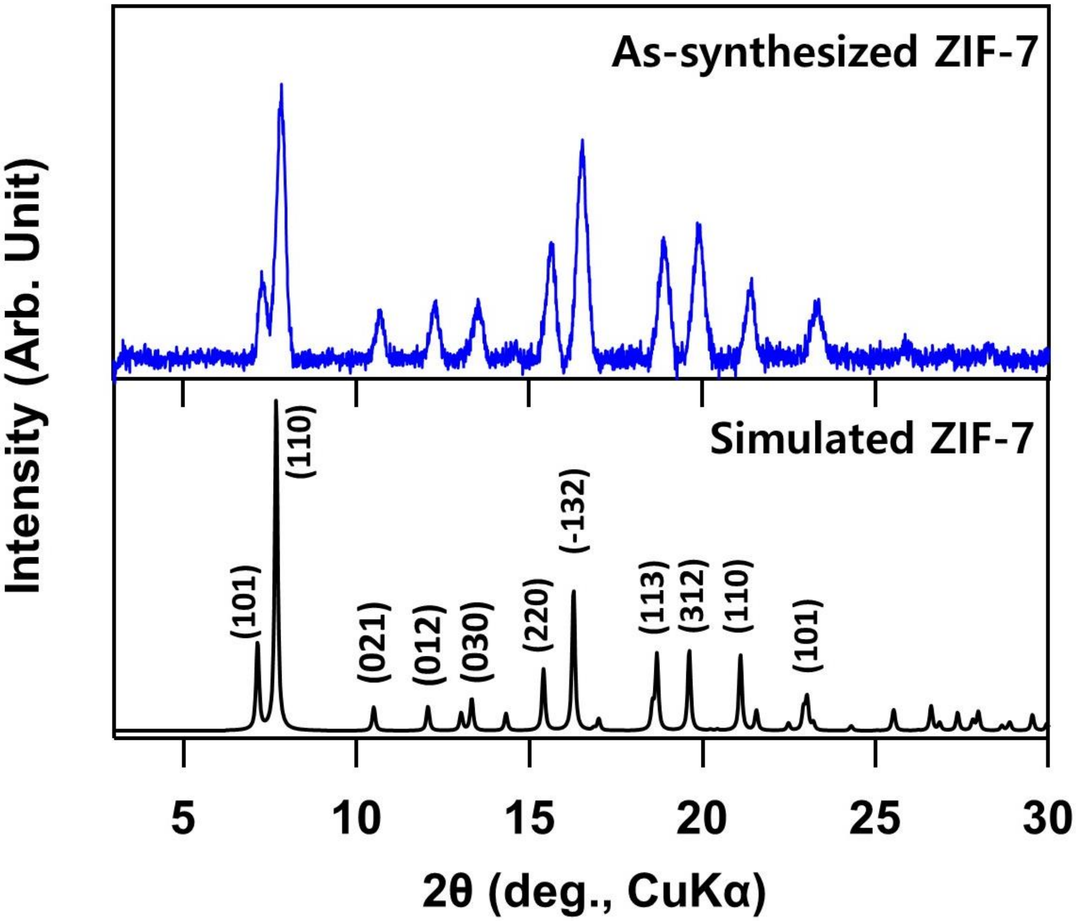

Supplementary Fig. 2 XRD patterns of as-synthesized ZIF-7 and Simulated ZIF-7.

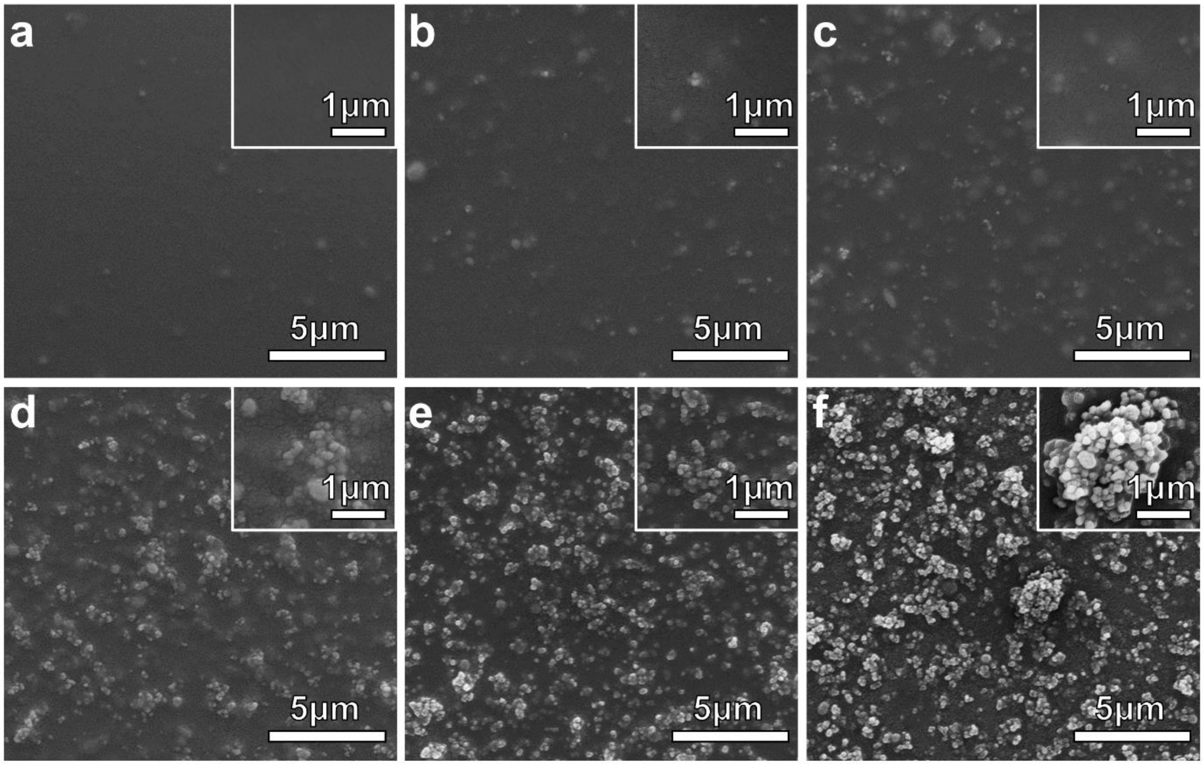

Supplementary Fig.3 Top-view SEM images of pure PEBA and MMM films. a pure PEBA film, **b** 2.5% ZIF-7/PEBA film, **c** 5% ZIF-7/PEBA film, **d** 10% ZIF-7/PEBA film, **e** 20% ZIF-7/PEBA film, and **f** 40% ZIF-7/PEBA film.

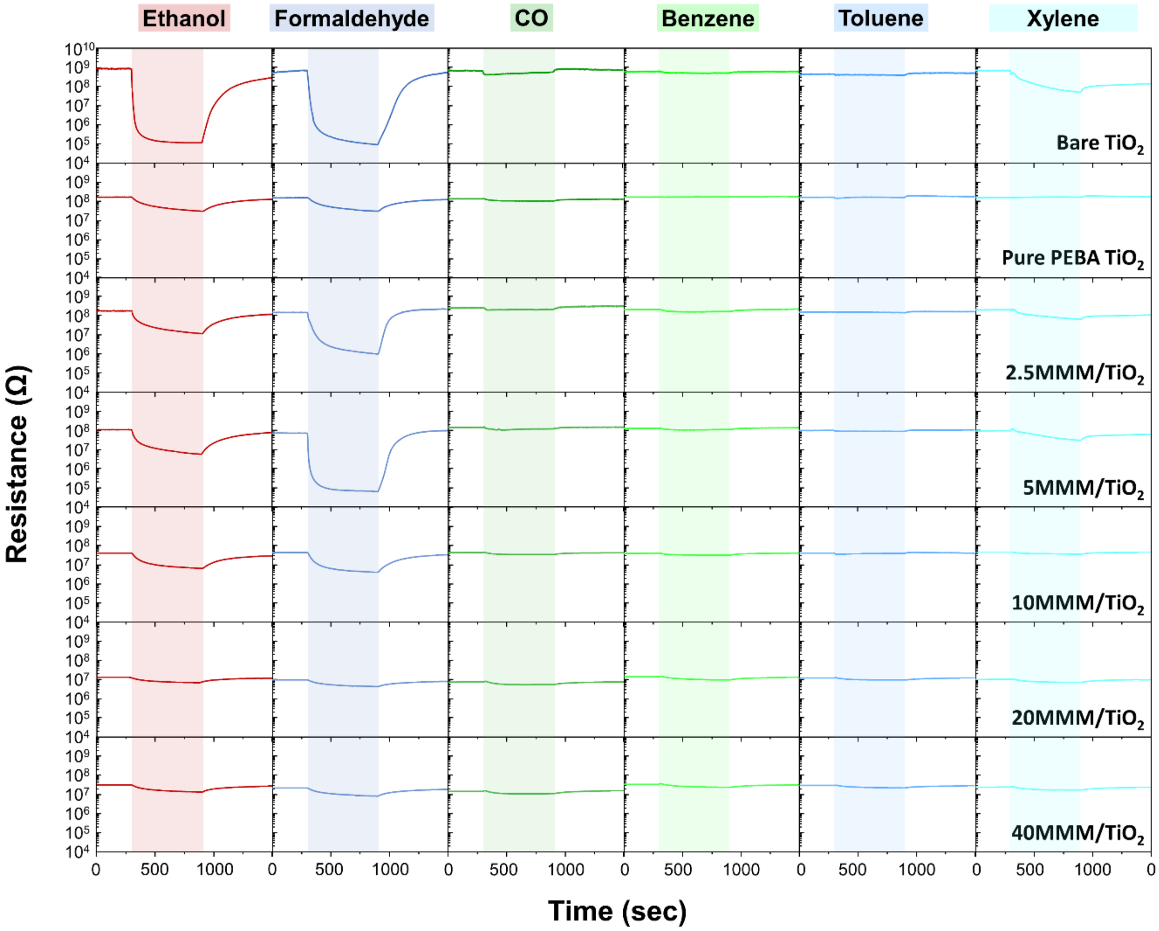

Supplementary Fig.4 Dynamic sensing transients of bare TiO_2 pure PEBA TiO_2, 2.5MMM/TiO_2, 5MMM/TiO_2, 10MMM/TiO_2, 20MMM/TiO_2, 40MMM/TiO_2 sensors (concentration of the analyte gas : 5 ppm; temperature: 23 °C; UV wavelength : 365 nm).

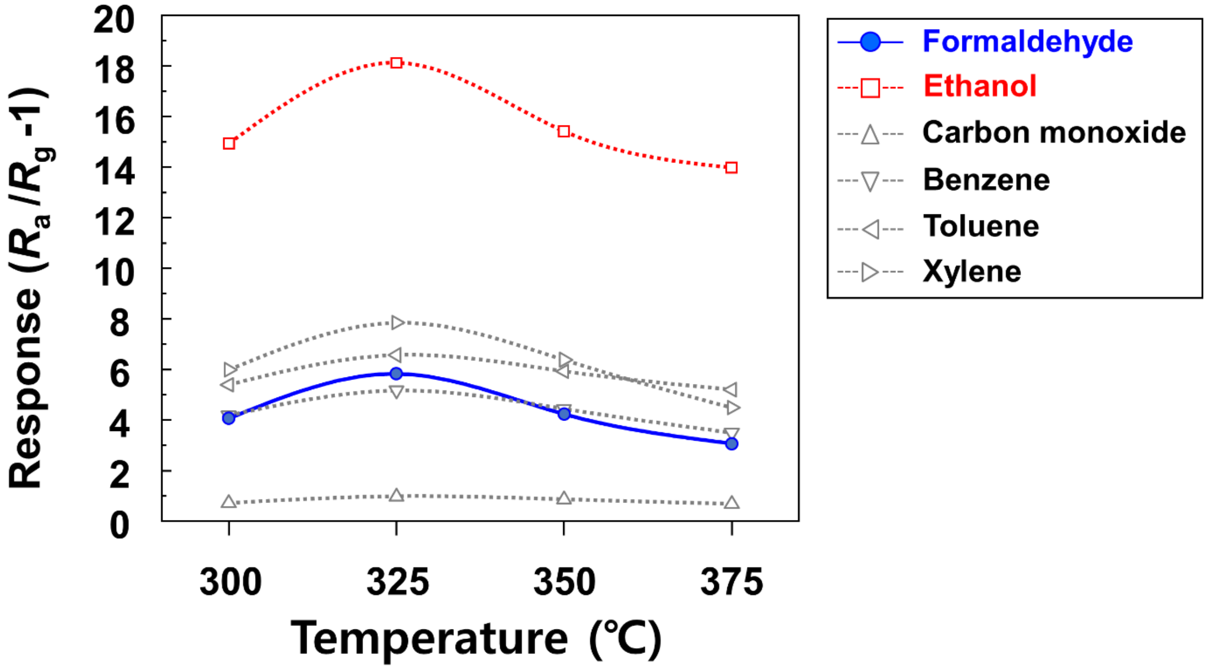

Supplementary Fig.5 Gas response of bare TiO_2 sensor to 5 ppm analyte gases. (temperature range : 300℃ ~ 375℃)

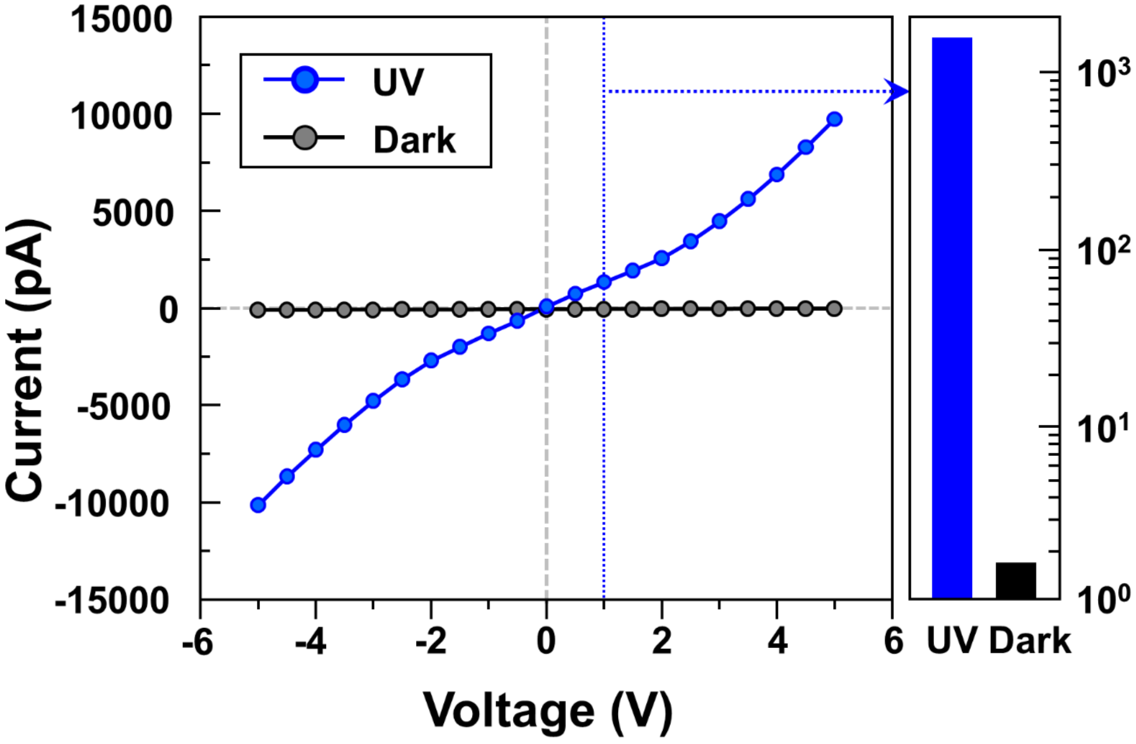

Supplementary Fig.6 I-V curve of TiO_2 under 365 nm UV and in dark condition. (UV wavelength : 365 nm; temperature: 23 °C)

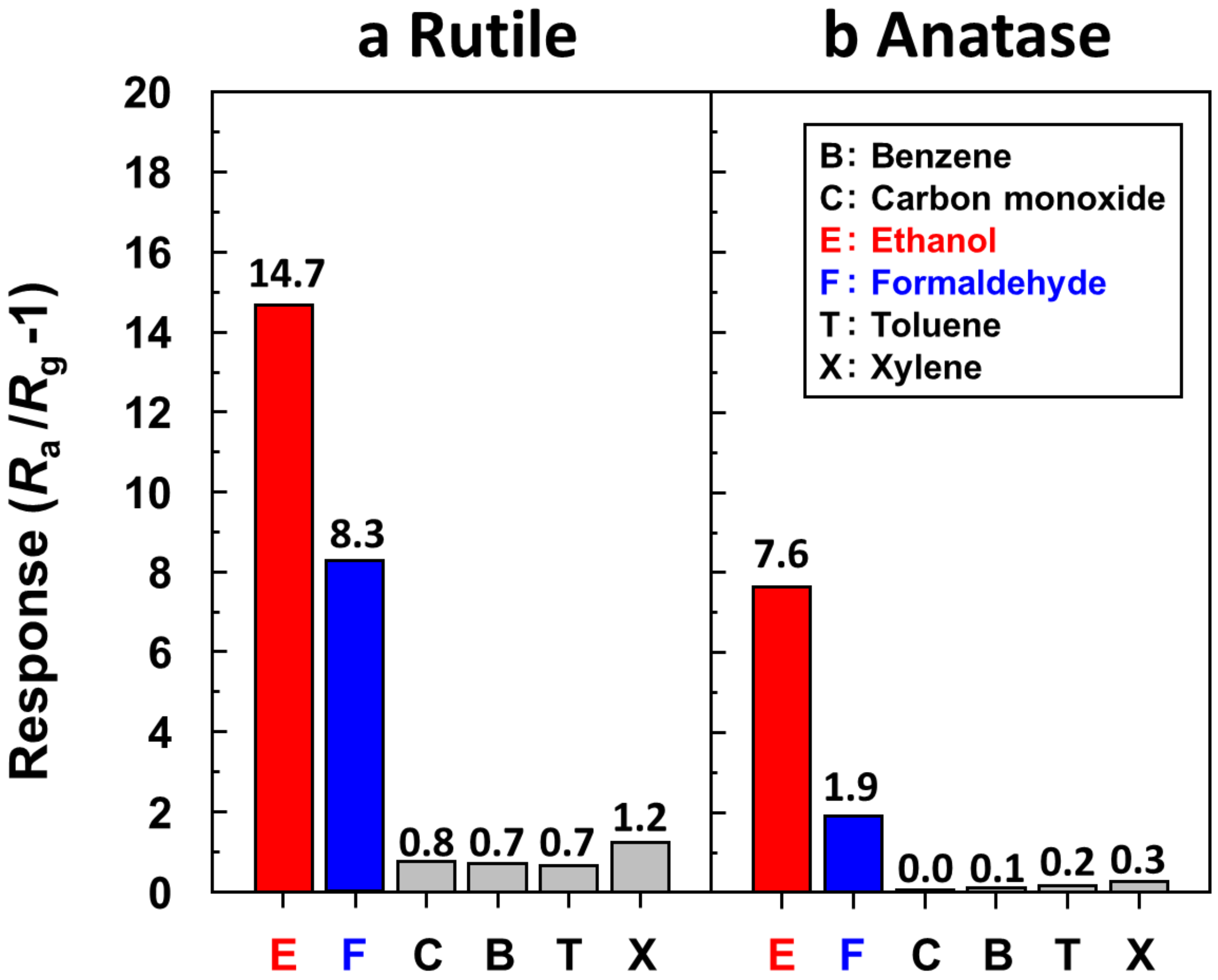

Supplementary Fig.7 Gas responses of rutile and anatase TiO_2 sensors. **a** rutile TiO_2, **b** anatase TiO_2 sensors. (concentration of the analyte gas : 5 ppm; ; temperature: 23 °C; UV wavelength : 365 nm)

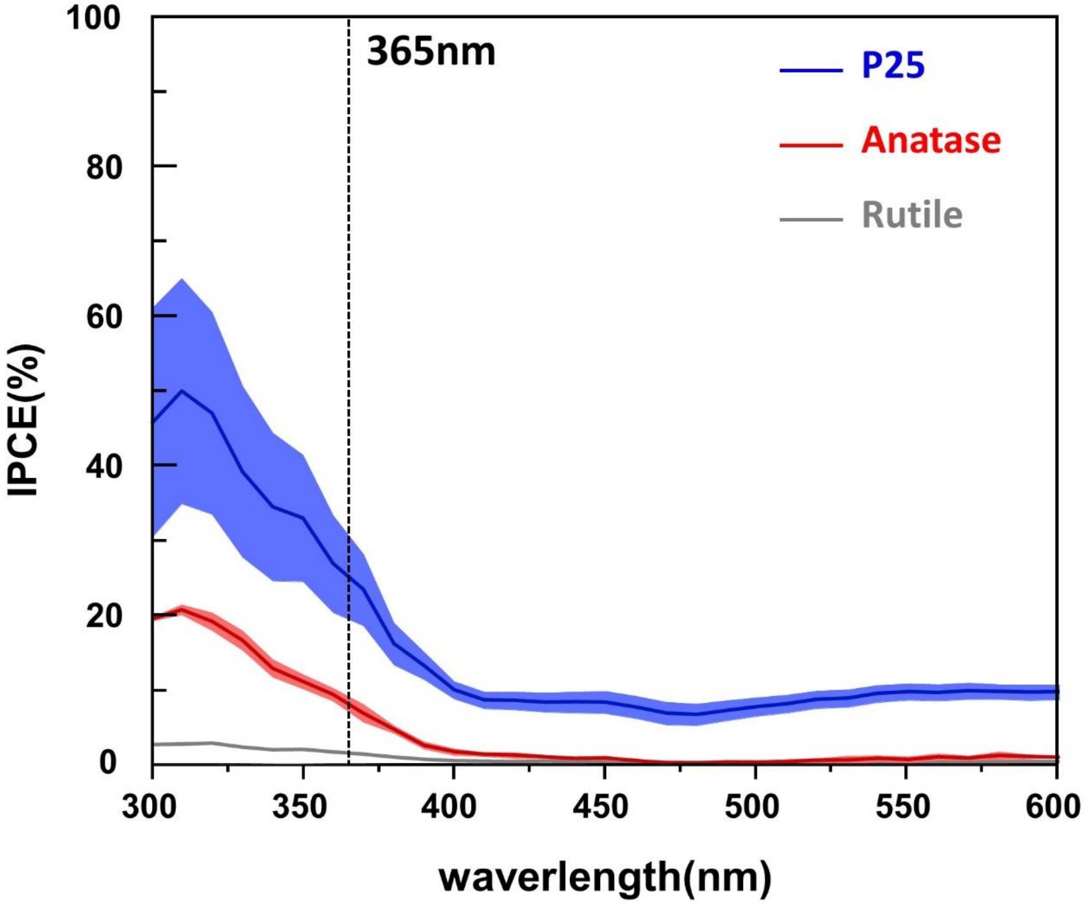

Supplementary Fig. 8 Incident photon-to-electron conversion efficiency (IPCE) of the P25(blue curve), Anatase(red curve), Rutile(black curve) in air atmosphere. Error bars represent s.d. of the mean.

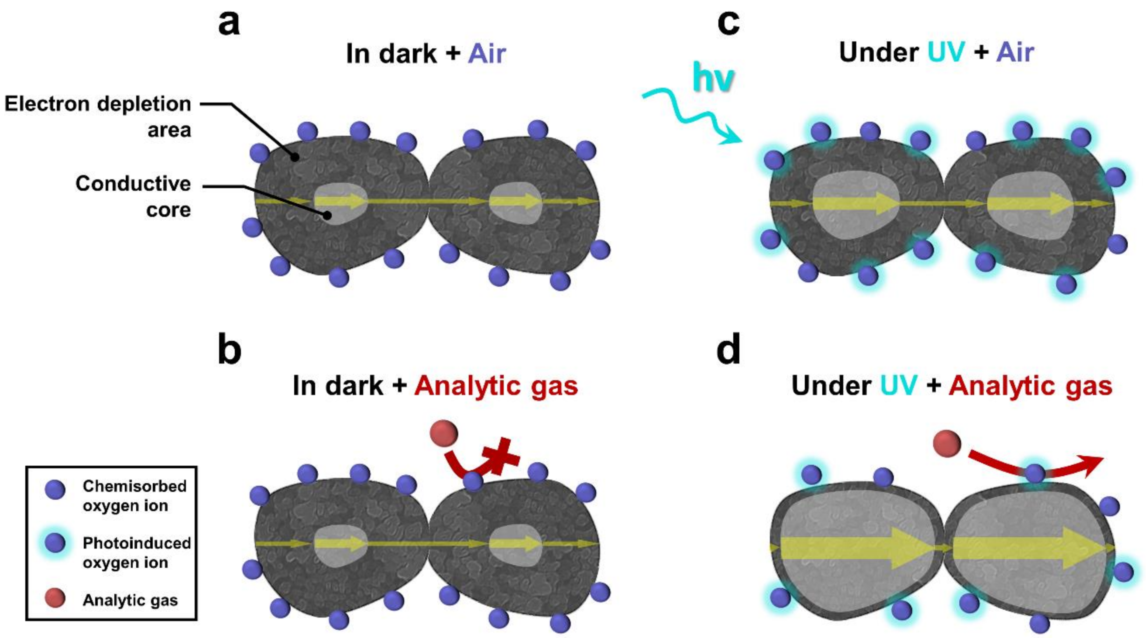

Supplementary Fig.9 Schematic illustration of mechanism of photo-assisted gas sensing under UV in room temperature.

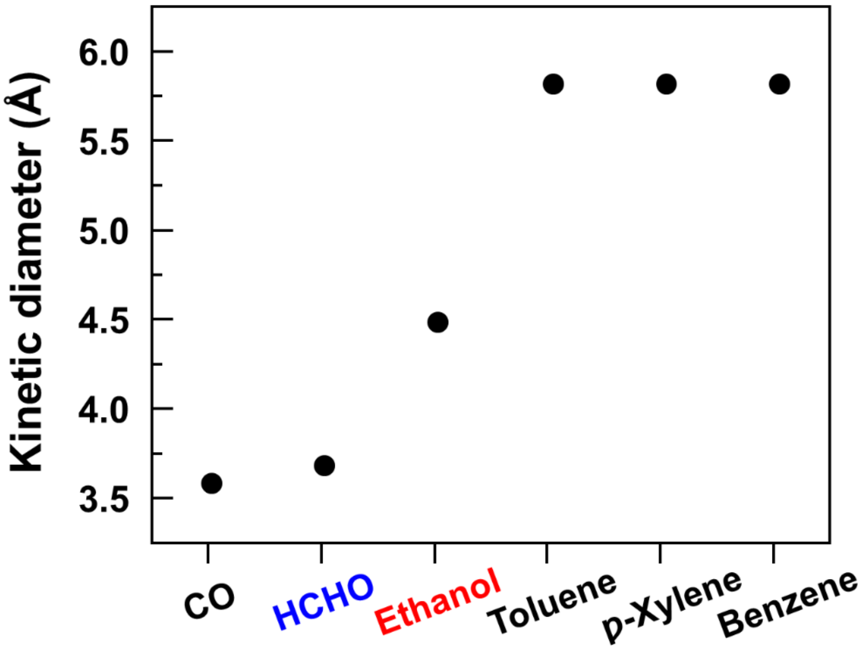

Supplementary Fig.10 Kinetic diameters of analyte gas.

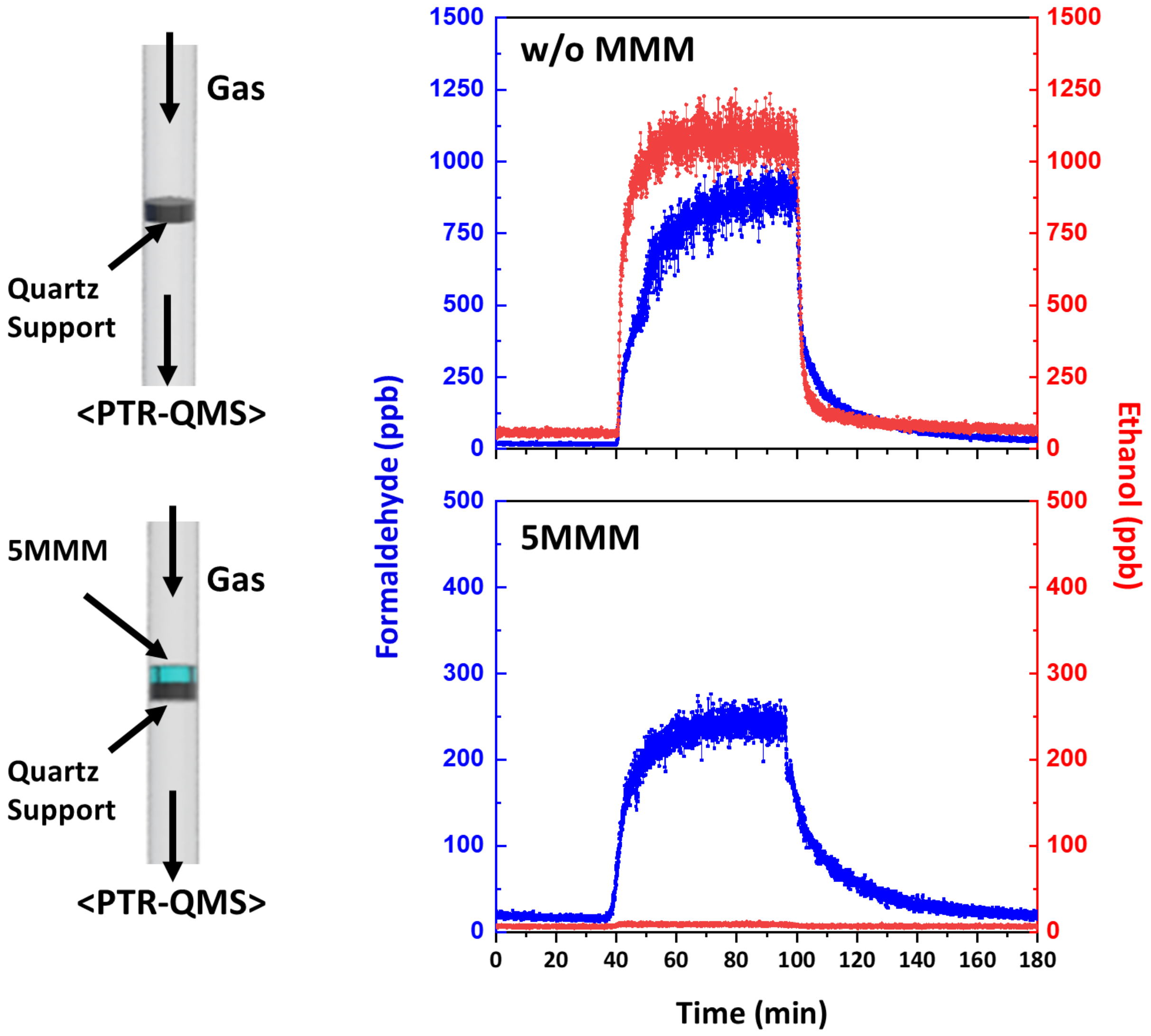

Supplementary Fig. 11 Outlet gas concentrations of formaldehyde and ethanol without membrane and those after 5MMM membrane (thickness: 1 mm) measured by PTR-QMS. (concentration of inlet mixture gas : 1 ppm formaldehyde + 1 ppm ethanol, flow rate 45 sccm, at room temperature)

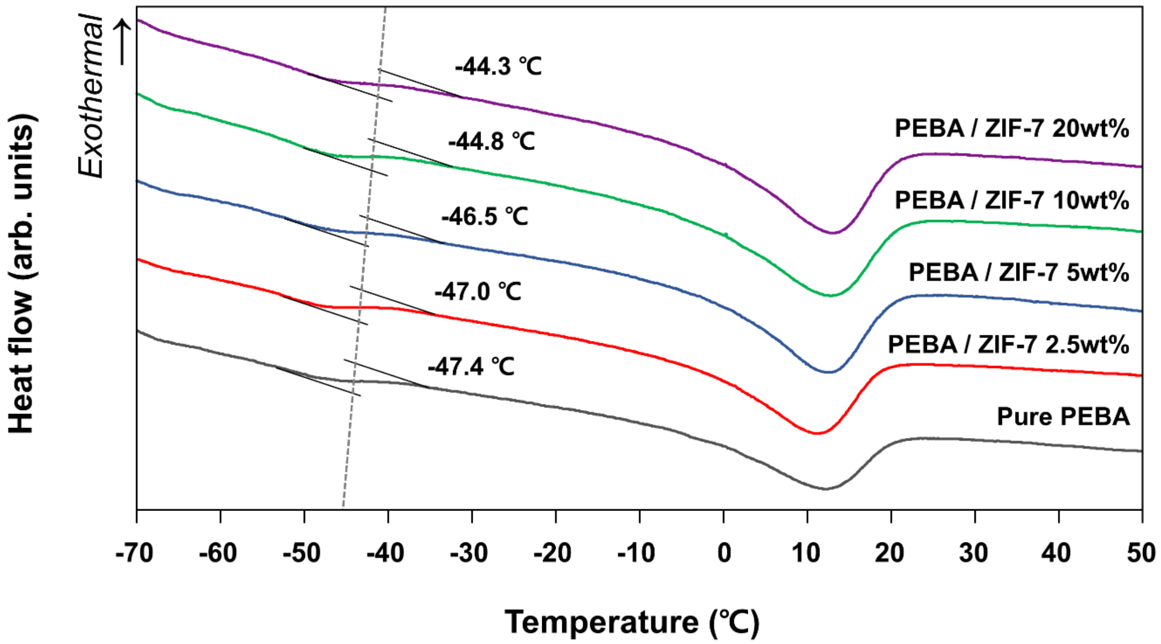

Supplementary Fig.12 DSC curves of the pure PEBA, 2.5MMM, 5MMM, 10MMM, and 20MMM.

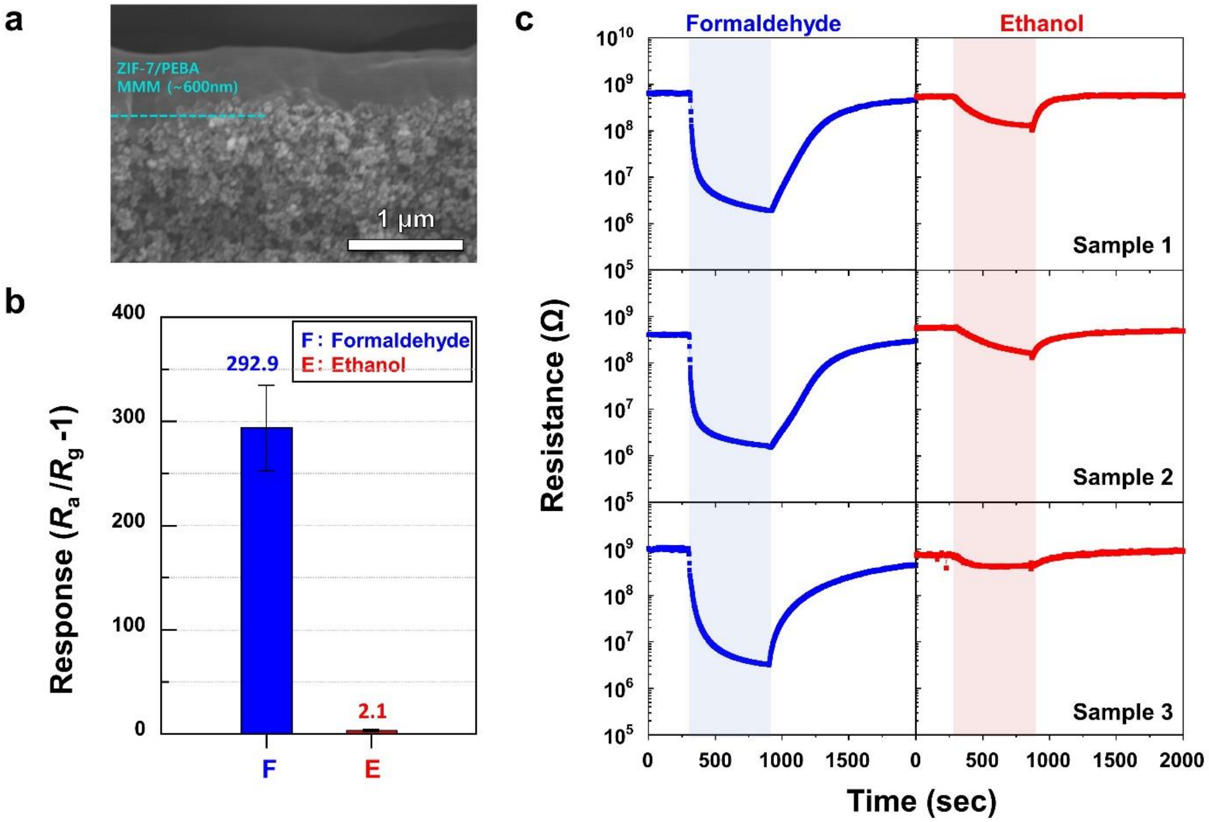

Supplementary Fig. 13 **a** SEM image of the 5MMM/TiO_2 sensor with the thick 5MMM layer (thickness: 600 nm) fabricated by 600 RPM spin coating. Error bars represent s.d. of the mean. **b** Gas responses to 5 ppm formaldehyde and ethanol. Error bars indicate the standard deviation of three measurements. **c** Dynamic gas-sensing transients to 5 ppm formaldehyde and 5 ppm ethanol. (temperature: 23 °C; UV wavelength: 365 nm)

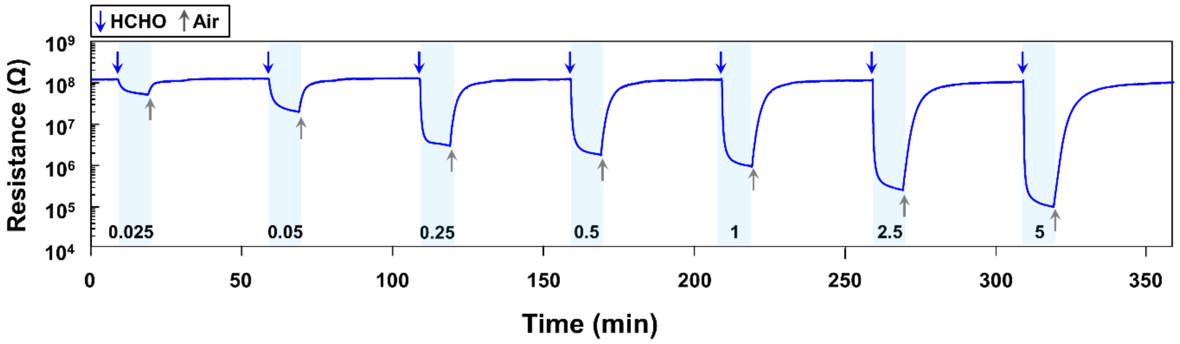

Supplementary Fig.14 Dynamic gas-sensing transients of the 5MMM/TiO_2 sensor to 0.025–5 ppm formaldehyde at 23 °C under 365 nm UV radiation.

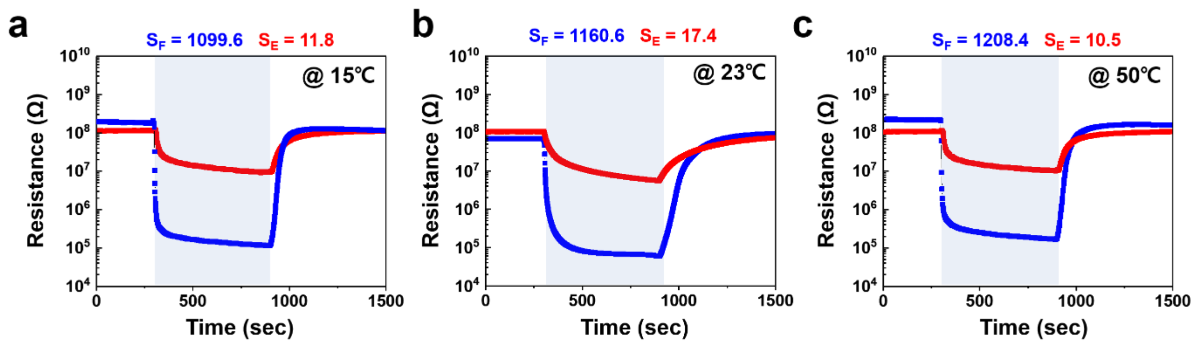

Supplementary Fig.15 Dynamic gas-sensing transients of 5MMM/TiO_2 sensor to 5 ppm HCHO and ethanol at **a** 15 ℃, **b** 23 ℃, and **c** 50 ℃ under 365 nm UV illumination.

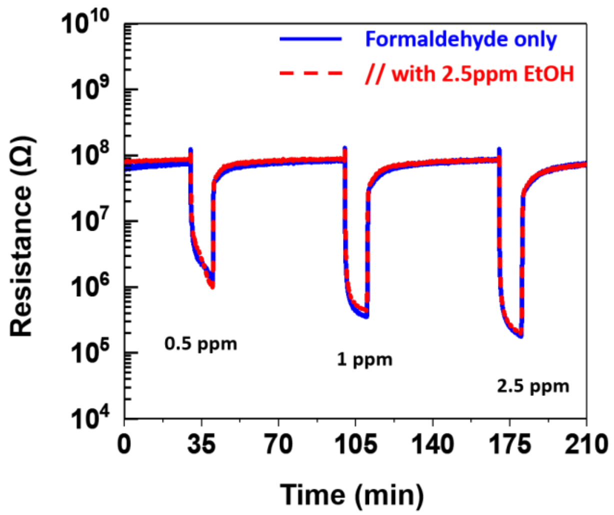

Supplementary Fig.16 Dynamic gas-sensing transients of 5MMM/TiO_2 sensor upon exposure to formaldehyde (0.5 – 2.5 ppm) (blue solid line) and mixture of formaldehyde (0.5 – 2.5 ppm) and ethanol (2.5 ppm) (red dashed line) at 23°C under 365 nm UV illumination.

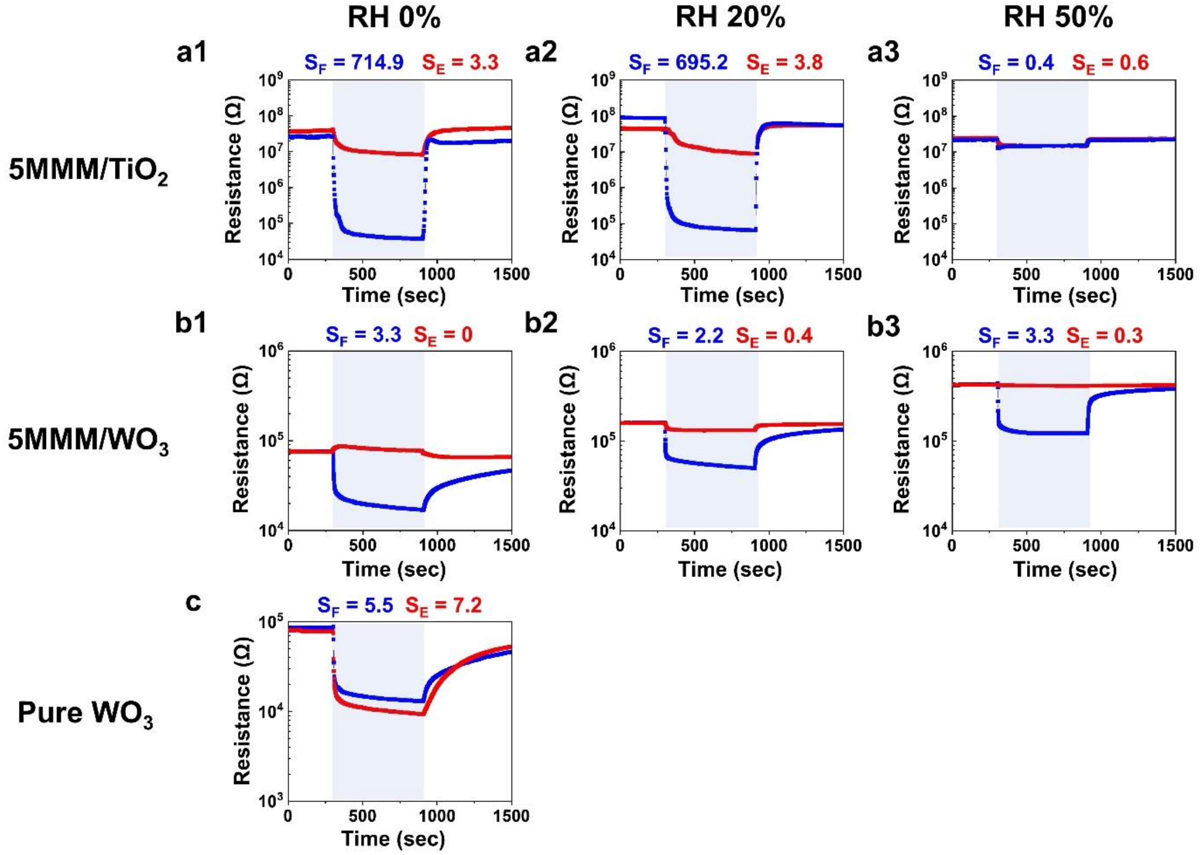

Supplementary Fig.17 Sensing transients of 5MMM/TiO_2, 5MMM/WO_3, and WO_3 sensors under various relative humidity. **a** 5MMM/TiO_2 sensor, **b** 5MMM/WO_3 sensor. **c** pure WO_3 sensor. (concentration of HCHO and ethanol: 2.5 ppm; temperature: 23 °C; UV wavelength: 365 nm)

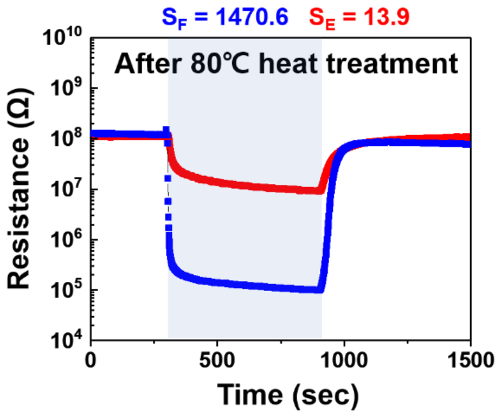

Supplementary Fig.18 Sensing transient of 5MMM/TiO_2 sensors to 5 ppm HCHO and ethanol under 365 nm UV illumination (operating temperature: 23 °C) after thermal annealing of the sensor at 80 °C.

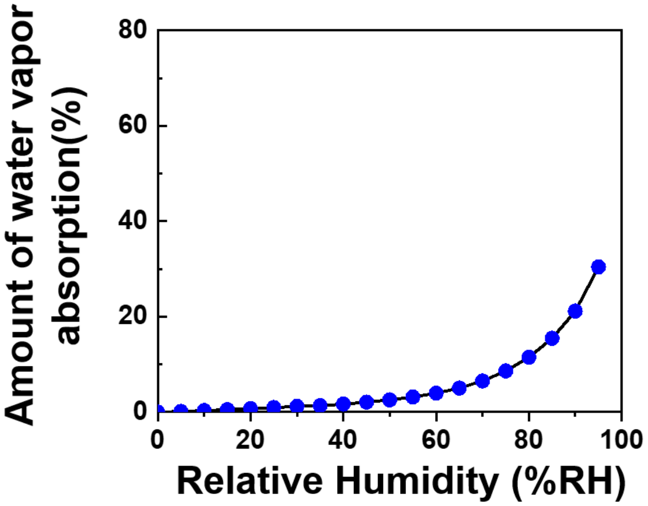

Supplementary Fig.19 Dynamic vapor sorption(DVS) of the 5MMM at 25 ℃.

Supplementary Table 1. Gas responses ($R_aR_g^{-1} - 1$, $R_gR_a^{-1} - 1$, $I_gI_a^{-1} - 1$) of various materials to formaldehyde, as reported in literature and obtained in the present study.[S1-S22]

Materials	Response @ 5ppm	Selectivity (@ x ppm)	Sensor temp.[°C]	Ref.
Co_3O_4/ZnO hollow spheres with nanoparticles	3	5 (@10 ppm)	160	1
VG/SnO_2 nanoparticles	0.05 (R_a/R_g)	5 (@5 ppm)	R.T.	2
Ga−In bimetallic oxide nanofibers	5	4 (@100 ppm)	150	3
Co_3O_4/ZnO core-shell nanofibers	2	2 (@100 ppm)	220	4
NiO/SnO_2 microspheres	3	4 (@100 ppm)	200	5
Ni-SnO_2 nanoparticles	20	4 (@100 ppm)	200	6
SnO/ SnO_2 nano-flowers assembled from ultrathin nanosheets	20	2 (@50 ppm)	120	7
PdAu decorated SnO_2 nanosheets	9	9 (@5 ppm)	110	8
NiO-SnO_2 microflowers	5	3 (@100 ppm)	100	9
Co-rich $ZnCo_2O_4$ hollow nanospheres	29	6 (@0.4 ppm)	225	10
SnO_2@rGO nanocomposites	3	4 (@100 ppm)	160	11
Zn_2SnO_4/SnO_2 hierarchical octahedral structure	8	5 (@100 ppm)	200	12
rGO/ZnSnO3 microspheres	6	5 (@10 ppm)	103	13
ZnO@ZIF−8 nanorod	12	5 (@100 ppm)	300	14
In_2O_3/Co nanorods	10	3 (@100 ppm)	130	15
GO/SnO_2 nanosphere	125	22 (@100 ppm)	60	16
In_2O_3 hierarchical architectures	1.5	7 (@100 ppm)	260	17
GO/SnO_2	6	3 (@100 ppm)	120	18
SnO2 nanofiber/nanosheets	18	5 (@100 ppm)	120	19
Ag-ZnO nanocomposites	11	2 (@100 ppm)	240	20
SnO_2 Nanosheets	15	5 (@10 ppm)	200	21
GO/TiO_2 nanosheet	4	3 (@10 ppm)	R.T.	22
5MMM/TiO_2 on SiO_2	1351	57 (@5 ppm)	R.T.	This work
5MMM/TiO_2 on flexible PET	576	70 (@5 ppm)	R.T.	This work

References

S1. Bai, S. et al. Surface functionalization of Co_3O_4 hollow spheres with ZnO nanoparticles for modulating sensing properties of formaldehyde. *Sens. Actuators, B* **245**, 359-368 (2017).

S2. Bo, Z. et al. Decoration of vertical graphene with tin dioxide nanoparticles for highly sensitive room temperature formaldehyde sensing. *Sens. Actuator, B* **256**, 1011-1020 (2018).

S3. Chen, H., Hu, J., Li, G.-D., Gao, Q., Wei, C.& Zou, X. Porous Ga−In bimetallic oxide nanofibers with controllable structures for ultrasensitive and selective detection of formaldehyde. *ACS Appl. Mater. Interfaces* **9**, 4692-4700 (2017).

S4. Gao, X., Li, F., Wang, R. & Zhang, T. A formaldehyde sensor: Significant role of p-n heterojunction in gas-sensitive core-shell nanofibers. *Sens. Actuators, B.* **258**, 1230-1241 (2018).

S5. Gu, C., Cui, Y., Wang, L., Sheng, E., Shim, J. -J. & Huang, J. Synthesis of the porous NiO/SnO_2 microspheres and microcubes and their enhanced formaldehyde gas sensing performance. *Sens. Actuators, B.* **241**, 298-307 (2017).

S6. Hu, J. et al. Enhanced formaldehyde detection based on Ni doping of SnO_2 nanoparticles by one-step synthesis. *Sens. Actuators, B.* **263**, 120-128 (2018).

S7. Li, N. et al. A low temperature formaldehyde gas sensor based on hierarchical SnO/ SnO_2 nano-flowers assembled from ultrathin nanosheets: Synthesis, sensing performance and mechanism. *Sens. Actuators, B* **294**, 106-115 (2019).

S8. Li, G. et al. Bimetal PdAu decorated SnO_2 nanosheets based gas sensor with temperature-dependent dual selectivity for detecting formaldehyde and acetone. *Sens. Actuators, B* **283**, 590-601 (2019).

S9. Meng, D. et al. Low-temperature formaldehyde gas sensors based on NiO_2 heterojunction microflowers assembled by thin porous nanosheets. *Sens. Actuators, B* **273**, 418-428 (2018).

S10. Park, H. J., Kim, J., Choi, N. -J., Song, H. & Lee, D. -S. Nonstoichiometric Co-rich $ZnCo_2O_4$ Hollow Nanospheres for High Performance Formaldehyde Detection at ppb Levels. *ACS Appl. Mater. Interfaces* **8**, 3233-3240 (2016).

S11. Rong, X. et al. Effects of graphene on the microstructures of SnO2@rGO nanocomposites and their formaldehyde-sensing performance. *Sens. Actuators, B* **269**, 223-237 (2018).

S12. Shu, S., Wang, M., Yang, W. & Liu, S. Synthesis of surface layered hierarchical octahedral-like structured Zn_2SnO_4/SnO_2 with excellent sensing properties toward HCHO. *Sens. Actuators, B* **243**, 1171-1180 (2017).

S13. Sun, J. et al. Hybridization of $ZnSnO_3$ and rGO for improvement of formaldehyde sensing properties. *Sens. Actuators, B* **257**, 29-36 (2018).

S14. Tian, H., Fan, H., Li, M. & Ma, L. Zeolitic Imidazolate framework coated ZnO nanorods as molecular sieving to improve selectivity of formaldehyde gas sensor. *ACS Sens*., **1**, 243-250 (2016).

S15. Wang, Z., Hou, C., De, Q., Gu, F. & Han, D. One-step synthesis of Co-Doped In_2O_3 nanorods for high response of formaldehyde sensor at low temperature. *ACS Sens*. **3**, 468-475 (2018).

S16. Wang, D. et al. Mesoporous Ultrathin SnO_2 nanosheets in situ modified by graphene oxide for extraordinary formaldehyde detection at low temperatures. *ACS Appl. Mater. Interfaces* **11**, 12808-12818 (2019).

S17. Wang, S. et al. Oxygen vacancies and grain boundaries potential barriers modulation facilitated formaldehyde gas sensing performances for In_2O_3 hierarchical architectures. *Sens. Actuators, B* **255**, 159-165 (2018).

S18. Wang, D. et al. Enhanced formaldehyde sensing properties of hollow SnO_2 nanofibers by graphene oxide. *Sens. Actuators; B* **250**, 533-542 (2018).

S19. Wang, D. et al. Constructing hierarchical SnO2 nanofiber/nanosheets for efficient formaldehyde detection. *Sens. Actuators, B* **283**, 714-723 (2019).

S20. Xing, X., Xiao, X., Wang, L. & Wang, Y. Highly sensitive formaldehyde gas sensor based on hierarchically porous Ag-loaded ZnO heterojunction nanocomposites. *Sens. Actuators, B* **247**, 797-806 (2018).

S21. Xu, R. et al. Ultrathin SnO_2 nanosheets with dominant high-energy {001} facets for low temperature formaldehyde gas sensor. *Sens. Actuators, B* **289**, 186-194 (2019).

S22. Ye, Z. et al. Room temperature formaldehyde sensor with enhanced performance based on reduced graphene oxide/titanium dioxide *Sens. Actuators, B* **223**, 149-156 (2019).
